# Causal language and strength of inference in academic and media articles shared in social media (CLAIMS): A systematic review

**DOI:** 10.1371/journal.pone.0196346

**Published:** 2018-05-30

**Authors:** Noah Haber, Emily R. Smith, Ellen Moscoe, Kathryn Andrews, Robin Audy, Winnie Bell, Alana T. Brennan, Alexander Breskin, Jeremy C. Kane, Mahesh Karra, Elizabeth S. McClure, Elizabeth A. Suarez, on behalf of the CLAIMS research team

**Affiliations:** 1 Department of Global Health and Population, Harvard T.H. Chan School of Public Health, Boston, MA, United States of America; 2 Carolina Population Center, University of North Carolina at Chapel Hill, Chapel Hill, NC, United States of America; 3 Division of Gastroenterology, Hepatology and Nutrition, Boston Children’s Hospital, Boston, MA, United States of America; 4 Department of Medical Ethics and Health Policy, Perelman School of Medicine, University of Pennsylvania, Philadelphia, PA, United States of America; 5 Unaffiliated, Philadelphia, PA, United States of America; 6 Friedman School of Nutrition Science and Policy, Tufts University, Boston, United States of America; 7 Department of Global Health, Boston University School of Public Health, Boston, MA, United States of America; 8 Department of Epidemiology, Boston University School of Public Health, Boston, MA, United States of America; 9 Department of Epidemiology, University of North Carolina Gillings School of Global Public Health, Chapel Hill, NC, United States of America; 10 Department of Mental Health, Johns Hopkins Bloomberg School of Public Health, Baltimore, MD, United States of America; 11 Frederick S. Pardee School of Global Studies, Boston University, Boston, MA, United States of America; Universidad de las Palmas de Gran Canaria, SPAIN

## Abstract

**Background:**

The pathway from evidence generation to consumption contains many steps which can lead to overstatement or misinformation. The proliferation of internet-based health news may encourage selection of media and academic research articles that overstate strength of causal inference. We investigated the state of causal inference in health research as it appears at the end of the pathway, at the point of social media consumption.

**Methods:**

We screened the NewsWhip Insights database for the most shared media articles on Facebook and Twitter reporting about peer-reviewed academic studies associating an exposure with a health outcome in 2015, extracting the 50 most-shared academic articles and media articles covering them. We designed and utilized a review tool to systematically assess and summarize studies’ strength of causal inference, including generalizability, potential confounders, and methods used. These were then compared with the strength of causal language used to describe results in both academic and media articles. Two randomly assigned independent reviewers and one arbitrating reviewer from a pool of 21 reviewers assessed each article.

**Results:**

We accepted the most shared 64 media articles pertaining to 50 academic articles for review, representing 68% of Facebook and 45% of Twitter shares in 2015. Thirty-four percent of academic studies and 48% of media articles used language that reviewers considered too strong for their strength of causal inference. Seventy percent of academic studies were considered low or very low strength of inference, with only 6% considered high or very high strength of causal inference. The most severe issues with academic studies’ causal inference were reported to be omitted confounding variables and generalizability. Fifty-eight percent of media articles were found to have inaccurately reported the question, results, intervention, or population of the academic study.

**Conclusions:**

We find a large disparity between the strength of language as presented to the research consumer and the underlying strength of causal inference among the studies most widely shared on social media. However, because this sample was designed to be representative of the articles selected and shared on social media, it is unlikely to be representative of all academic and media work. More research is needed to determine how academic institutions, media organizations, and social network sharing patterns impact causal inference and language as received by the research consumer.

## Introduction

Clinical practitioners, policy makers, households, and all other health decision makers make choices based on their understanding of the evidence generated by scientific output. Many decision makers receive that information through traditional and social media, with an estimated 62% of Americans having received news through some form of social media in 2016 [1]. The pathway from research generation to consumption begins with academic research production, followed by publication, then media reporting, and ultimately leading to distribution on social and traditional media. Each step contains many mechanisms which could yield inaccurate and/or overstated evidence at the point of media consumption. Because each step of this pathway builds upon the previous one, issues with systematic selection, spin, overstatement, inaccuracy, and weak evidence are likely to accumulate by the time scientific research reaches the consumer.

Recent evidence suggests that exposure to health information in traditional and social media may impact health behaviors [2]. An example in the UK [3] highlights the dangers of weak and poorly-reported evidence: An estimated 200,000 patients temporarily ceased taking statins–drugs with strong evidence for efficacy and safety for treating hypertension [4, 5]–after press outlets reported on two studies suggesting that the medication was associated with high rates of adverse events. The authors of both publications later made statements retracting their conclusions, noting that they had overstated the strength of causal inference in light of the studies’ lack of methodological rigor [3]. Further contributing to the cycle, media coverage of specific conditions is associated with increased reporting of adverse events [6], public announcements of celebrity illnesses are associated with surges in screening and diagnostic tests [7, 8], and language “spin” may influence clinical decision makers’ assessment of evidence strength [9].

We hypothesize that incidents like the example of statin use in the UK are the culmination of selection and spin at each step on the pathway from research generation to research consumption. Scientific health studies are created in academic institutions. Some are selected for publication in scientific, peer-reviewed journals. A subset of these are reported on by traditional media outlets, and the most popular reports are shared on social media. Feedback may exist where the expectation of selection and spin in later stages influences decisions at earlier steps in the pathway. Incentives for research institutions, journals, media outlets, and consumers may combine to create an environment where inaccurate or overstated conclusions are elevated and amplified by the time scientific research reaches the consumer.

A scientist’s academic success is often tied to research and publications. Producing high-impact work is crucial for career continuity and advancement, measured primarily through the number of times it is cited and the impact factor of the journal in which it is published. These incentives apply across the academic spectrum, from individual researchers, to research intuitions, to press releases [10–12], and to peer reviewed publication [13, 14]. A host of factors can render studies inaccurate [15] or not useful in practice [16]. From a methodological perspective, there are many challenges to making strong causal inference in human populations. At the individual study level, internal validity may be weak due to uncontrolled confounding variables, improper use of statistical methods, and use of cherry-picked data and methods to achieve statistically significant results [17]. External validity may be threatened by the limited generalizability of study populations to relevant populations or differences between the study environment and real-world interventions [18, 19]. Publication bias [20, 21], lack of replicability and replication [22], implication of a causal relationship even after stating that the evidence is insufficient to reach causal conclusions [23–25], and related phenomena may also introduce error into bodies of published literature.

Traditional and social media are changing the way many audiences consume science. Media outlets may spin [26–28] and encourage dissemination of eye-catching, potentially overstated, inaccurate [29], and/or misleading headlines in order to gain larger audience size [28, 30, 31]. Media companies’ revenues rely on advertising [1, 32] which may put them at odds with the journalistic values of media rigor and objectivity, and may incentivize the production of potentially inaccurate health news that is not commensurate with the evidence. The interaction between academia and media highlights some of these complexities, such as a recent study finding that the results of over half of studies on an association between exposure and outcome covered in the news are refuted by subsequent meta-analyses [33], and another showing that those articles publicly critiqued in online media are more likely to be retracted [34].

This study examines the state of causal inference in health research as it reaches the consumer–the endpoint of the research pathway–by systematically reviewing the articles that are most likely to have been shared on social media. We argue that the academic research publications comparing an exposure and health outcome and media articles covering them that reach the public should at minimum have language that matches the strength of their causal inference, with a preference for studies demonstrating stronger causal inference.

Based on these principles, the objectives of this study were to identify the media articles and related academic literature measuring the association between an exposure and a health outcome most shared through social media in 2015 and assess 1) the strength of causal inference in research articles from scientific journals, 2) concordance between strength of inference and the use of causal language in those articles, and 3) the strength of causal language used in corresponding media articles.

## Methods

The study was designed to identify and review causal inference and language from the 50 most shared academic articles pertaining to single studies of an exposure and a health outcome. We used Facebook and Twitter social media sharing statistics generated from the NewsWhip Insights platform to achieve a representative sample of the research to which the general news-consuming public is most likely to be exposed. We reviewed the academic research articles mentioned in the news articles for strength of causal inference followed by the appropriateness of the causal language used in both the original academic study and the media article(s) reporting on that research. While this scheme generates a sample that is representative of public consumption and distribution, it is neither intended nor likely to be representative of the total literature produced by academia and media.

The final protocol for this project was developed and reported using PRISMA guidelines for systematic review [35], and registered with PROSPERO [36] as protocol CRD42016045197 on August 5, 2016. The PROSPERO-registered protocol is attached as S2 File. The full protocol, which contains additional detail on the selection and review process, can be found at https://www.metacausal.com/CLAIMS/protocol/.

### Search strategy and selection criteria

We obtained a dataset of potential social media news articles for this study from the NewsWhip Insights™ platform [37]. The NewsWhip Insights platform is a privately-operated social media crawler, which has been collecting data since 2014. The platform identifies media “stories” (article URLs) and tracks how they are distributed on social media platforms. We queried the NewsWhip Insights dataset to generate a list of health news articles pertaining to new research studies published in 2015 (published from January 1, 2015 to December 31, 2015), querying the database on May 3, 2016. The search terms for this query were:

(categories:2) AND ((headline_en:"health" OR summary_en:"health") AND ((headline_en:"study" OR summary_en:"study") OR (headline_en:"research" OR summary_en:"research")))

where *categories*:*2* corresponds to NewsWhip’s internally curated categorization for sites containing news, *headline_en* is the programmatically extracted headline of the article at that URL, and *summary_en* is the programmatically generated content in English.

We defined the “popularity” of a media article as the number of times a URL was shared on Twitter or Facebook, where Twitter Tweets/Retweets and Facebook posts containing the URL are each considered a share. “Likes,” “favorites,” and comments are not considered shares for our analysis. We generated a list of the top 1,000 most shared health article URLs in 2015 from each social network, giving each a randomized numerical identifier, and sorting from most to least shared on its network. We combined the Twitter and Facebook datasets into a single merged dataset roughly equally representing each social network. We started with the most shared media article URL on a randomly selected social network (Twitter), selecting that article into our screening sample, and eliminating it from the Facebook list. The procedure was then repeated starting from the remaining list of articles from Facebook (eliminating the article from the Twitter list), and repeated, switching lists at each step.

To determine eligibility for inclusion in our study, we screened both the media article and academic article to identify media articles reporting on a single identifiable academic study about the relationship between an exposure and health outcome. The full inclusion criteria are outlined in Table 1. Two independent researchers reviewed the media article and the corresponding academic article titles and abstracts to determine whether they met the criteria for inclusion in the study. A third reviewer reconciled any disagreements between reviews and made a final decision regarding inclusion or exclusion. We reviewed the articles in order of number of shares, beginning with the most shared and continuing until there were 50 unique academic articles eligible for inclusion in the study. Each academic article and its set of associated media articles were considered a single review unit for the next phase.

**Table 1:** Inclusion criteria.

Media article:	Academic article:
• The URL link to media article is functional at the time of the review, leading to the main media article. • The news media article reports primarily about the findings from a single academic article published in a peer-reviewed academic journal. • The news media article reports that the academic article: - Has a main analysis in the form exposure (dependent variable) vs. outcome (independent variable). - Has a health outcome as one of its main outcomes (independent variables). - Has related exposures/outcomes if multiple exposures/outcomes are equally emphasized. - Conducted the study in a human population. - Presents results based on primary data analysis, and not a review or meta-analysis. - Has main results generated from a single identifiable statistical model.	• The academic article referred to in the media article is identifiable through academic library sources. • The academic article - Has a main analysis of the form exposure (dependent variable) vs. outcome (independent variable). - Has a health outcome as one of its main outcomes (independent variables). - Has related primary exposures/outcomes if multiple exposures/outcomes are equally emphasized. - Conducted the study in a human population. - Presents results based on primary data analysis, and not a review or meta-analysis. - Has main results generated from a single identifiable statistical model.

### Data collection

Each article was reviewed by two independent, randomly-assigned, primary reviewers from our study reviewer pool. The primary reviews were then given to a randomly-assigned arbitrating reviewer. The arbitrating reviewer created the final review, using the two independent reviews as guidance. The arbitrating reviewer was given the opportunity to ask clarifying questions to the primary reviewers using a standardized form. All reviewers served as both primary and arbitrating reviewers. The identities of each reviewer were and remain fully anonymous to each other and the public through a combination of the use of random-digit identifiers and the study lead acting as the go-between administrator for all between-reviewer communication. The reviews were performed under a strict policy of independence. The study lead was not a reviewer of the articles, and did not answer questions or give advice pertaining to any particular review article. All reviewers were given opportunity to recuse themselves from reviewing each assigned article in cases of perceived financial or social conflicts of interest with article authors and/or for lack of sufficient methodological or content area knowledge.

### Reviewers

Twenty-one reviewers were recruited through peer reference and public notices. All reviewers had completed at least a master’s degree and/or were currently enrolled in a doctoral degree in a relevant health and/or quantitative sciences field, with documented coursework relating to quantitative causal inference, with the large majority (n = 19/21) currently pursuing or having completed a relevant doctoral degree. Reviewers performed work on a purely voluntary basis with no financial incentives. The list of reviewers is published; however, the specific review assignment for each article is confidential.

### Review tool

The authors designed, tested, and implemented a review tool for this study to systematically assess the strength of causal inference in academic articles, the strength of causal language used by academic study authors, the strength of causal language used by media article authors, and the accuracy of the media article’s description of the academic article. We developed this tool using the principles outlined by the Cochrane Handbook for Systematic Reviews of Systematic Reviews of Interventions [38] and the Oxford Centre for Evidence-Based Medicine Levels of Evidence [39]. Existing tools typically focus on literature from randomized controlled trials and/or seek to summarize the strength of causal inference from a body of literature on the same subject. The review tool used for this study instead seeks to examine the strength of causal inference in individual studies. The full review tool questionnaire items are included in S1 File, with the original form interface used available at metacausal.com/claims/review-tool/.

**Fig 1:**
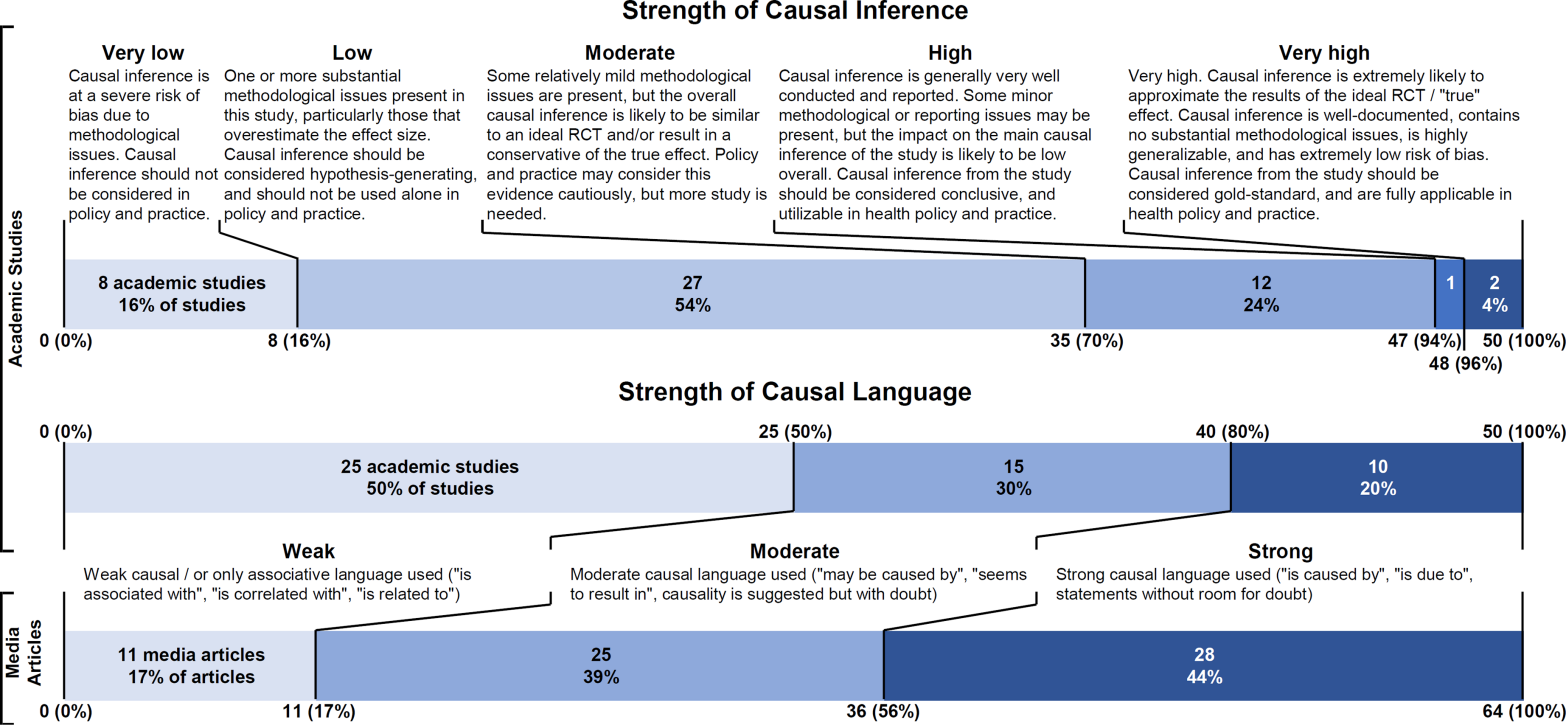
Results of summary measures for strength of causal inference and language. Language for categories of strength of causal inference has been lightly edited for this publication to better reflect the instructions given to the reviewers and for consistency with the rest of the manuscript. Reviewers were instructed to consider only the causal inference aspect of the study for these measures. The original language referred to the “study” and “results,” which has been edited in the figure to “causal inference” where appropriate for clarity. The original language and instructions are available in the attached review tool and on metacausal.com.

The largest section of the review tool assessed the scientific article’s strength of causal inference, focusing on risk of bias, considering both internal and external validity. The tool was divided into several domains which assessed the primary study question(s), primary study result(s), generalizability to a relevant population, selection bias/missing data, exposure and outcome assessment, treatment of covariates and identification of confounders, assessment of statistical methods, and a summary assessment. The summary assessments asked whether the study was likely to approximate hypothetical results from an “ideal” randomized controlled trial which would result in perfectly estimated and generalizable causal effects without practical and ethical constraints [40]. After completing each of the previous sections, reviewers assessed a summary measure of the severity of threats to causal inference in each of the above-listed subsections on a 5-point scale (Very low, Low, Moderate, High, Very high severity), as well as the expected direction of bias. The final assessment of this section summarizes the reviewer’s overall evaluation for the strength of causal inference on the same 5-point scale (Very low, Low, Moderate, High, Very high strength) with detailed prompts on which to base a decision. We assessed the strength of causal language in the scientific article on a 3-point scale (Weak, Moderate, Strong) based on the degree of certainty of causal inference implied, similar to the scale used in Brown, et al., 2013 [41] and Sumner et al., 2014 [12]. Two elements of language were assessed using this scale: how the authors framed their question of interest and how the authors discussed their results. The prompts for both summary sections are shown in Fig 1. In the media article assessment, reviewers assessed whether the media article accurately described the same relationship as the scientific article and the strength of causal language used, using the same research question as in the scientific article. For both academic and media articles, reviewers were asked whether they believed the strength of causal language was too weak, accurately descriptive, or too strong given the strength of causal inference.

The user interface for the review tool was developed using Google Forms (www.google.com/forms) and was implemented as an online survey.

### Statistical analysis

Statistical analysis is performed by descriptive statistics of the proportion of articles reaching each category for summary measures. Statistical analysis and data management was performed in R [42]. Two-tailed t-tests were used for statistical significance for differences in means.

### Publicly available data

The review dataset (S4 File) and review tool (S1 File) are provided as supplementary attachments to this study. In addition, the full protocol, review tool, screening process summary, full dataset collected from both primary and arbitrating reviewers, R code used to generate the dataset and analyses, and data visualization tools to explore the dataset are publicly available at metacausal.com/CLAIMS.

## Results

We identified 11,349 unique URLs in the NewsWhip database, which represented 1,375,152 shares on Facebook and 423,996 shares (Tweets) on Twitter. We extracted the 1,000 most shared articles from both platforms (n = 2,000) and consolidated them into a master article list comprised of 1,418 non-duplicate links to articles. Two independent reviewers screened the master list, starting from the most popular articles and continuing until 50 unique academic articles and associated media articles were identified, which occurred on the 231^st^ media article. The cumulative number of shares among the 231 screened articles screened represents 68% (938,596/1,375,152) of Facebook shares and 45% (189,777/423,996) of Twitter shares (S1 Fig), suggesting that our sample is strongly representative of the articles most shared on social media. Reviewers disagreed on whether to accept or reject in 29/231 cases, and the arbitrator included 16 of these cases. In total, reviewers identified 50 review sets, consisting of 50 academic articles with 64 unique media articles associated with them (S2 Fig, S2 and S3 Tables).

Reviewers were randomly assigned as a primary reviewer or arbitrator to an average of 7.1 articles each, and review work was completed between August and October, 2016. Reviewers opted to recuse themselves in nine instances; five recusals were due to conflicts of interest and four were due to lack of familiarity with the methods used in the academic article. Arbitrators made comments or asked for clarification from primary reviewers for 22 article sets. All results below, unless otherwise noted, are generated from the final arbitrator reviews alone.

Of the 50 academic articles reviewed, 49 listed at least one author affiliation with at least one primarily academic institution, 30% with a health service provider, 28% with a government agency, 24% with a non-profit organization, and 8% with a for-profit affiliation, as determined by the study authors. Authors from 192 unique institutions contributed to these 50 academic articles, with researchers from Harvard University (n = 8, 16%) and Johns Hopkins University (n = 4, 8%) being the most common, where n refers to the number of academic articles in which at least one author was listed as an affiliate. Academic journals in our sample consisted of many of the highest impact factor medical journals for 2015, with impact factors ranging from 2–60 [43]. The most commonly shared media URL domains were CBS News (n = 6), The Guardian (n = 6), and the New York Times (n = 6) (S1 Table).

Descriptive statistics of included academic studies are shown in Table 2. 14% (n = 7) of studies were randomized trials, while 44% (n = 22) were prospective cohorts, 26% (n = 13) cross sectional, and 12% (n = 6) other observations designs. The median sample size was 5,143.5. The most common exposures of interest were the built urban/rural environment (n = 5, 10%), diet (n = 5, 10%), coffee/caffeine (n = 4, 8%), medical device/treatment (n = 4, 8%), and pregnancy/childbirth (n = 4, 8%). The most common outcomes of interest were mood or mental health (n = 10, 20%), cardiovascular disease (n = 7, 14%), cognitive functioning or schooling (n = 5, 10%), and mortality (n = 5, 10%).

**Table 2:** Academic article descriptive statistics.

*Panel a*: *Study properties*				
	**Study type**	% (n)	**Methods**	% (n)
	Randomized controlled trial	14% (7)	Standard correlation	80% (40)
	Standard RCT	86% (6)	Hierarchical/longitudinal	20% (10)
	Crossover trial	14 (1)	Instrumental variable	2% (1)
	Observational	82% (41)	Marginal structural model	2% (1)
	Prospective cohort	54% (22)	Can't be determined	2% (1)
	Cross-sectional	31% (13)	Other	10% (5)
	Case-control	5% (2)		
	Retrospective cohort	2% (1)	**Sample size**	n
	Ecological	2% (1)	25th percentile	326.5
	Other	5% (2)	Median	5,143.5
	Other	4% (2)	75th percentile	34,849
*Panel b*: *Exposures and outcomes*				
** **	**Exposures**	% (n)	**Outcomes**	% (n)
** **	Built environment	10% (5)	Mood / Mental health	20% (10)
** **	Diet	10% (5)	CVD	14% (7)
** **	Coffee / Caffeine	8% (4)	Cognitive function / Schooling	10% (5)
** **	Medical device / Treatment	8% (4)	Mortality	10% (5)
** **	Pregnancy / Delivery	8% (4)	Self-rated Health	8% (4)
** **	Pet / Animal-related	6% (3)	Weight/BMI	8% (4)
** **	Race / Ethnicity / Sex / Gender	6% (3)	Blood biomarkers (multiple)	4% (2)
** **	Air pollution	4% (2)	Cancer	4% (2)
** **	Marriage / Partnership / Children	4% (2)	HIV	4% (2)
** **	Mindfulness / Meditation / Yoga	4% (2)		
** **	Other	32% (16)	Other	18% (9)

Data from panel a directly reflects the categorizations of study types and methods reviewers were given in the review tool, where the results shown are the arbitrator-determined categorizations. Additional details on the categories are available in the review tool itself, provided as a supplement, and in the Review Tool section of this manuscript. Panel b reflects categories determined post-hoc by the study authors, given the arbitrator-reported exposures and outcomes. The uncategorized reviewer-listed outcomes and exposures are provided in the publicly available datasets.

More than half of the studies (n = 27, 54%) were rated as having “Low” strength of causal inference, and eight studies (16%) were classified as “Very low” strength of causal inference. Only three studies (6%) were classified as having “High” or “Very high” strength of causal inference (Fig 1). Nearly half of the media articles (n = 28, 44%) were rated as having used “Strong” strength of causal language compared with 20% (n = 10) of the academic articles. Half (n = 25) of the academic articles used were rates as having “Weak” strength of causal language, compared with only 17% (n = 11) of the media articles.

Reviewers generally gave qualitatively similar ratings for each article. Primary reviewers differed in their rating of strength of causal inference by at most one or zero categories in 96% of cases, with 58% of all pairs of primary reviews answering with the exact same category. Mean arbitrator reviews, treating each category as consecutive integers from 1:5, where 1 = “Very low” and 5 = “Very high”, were only slightly lower than the mean arbitrator reviews (2.24 vs. 2.30, p-value = 0.71). 93% of primary reviewers chose within one category of each other for strength of causal language in the academic studies, with 47% having exact matches, noting there were only three categories for this measure.

**Fig 2:**
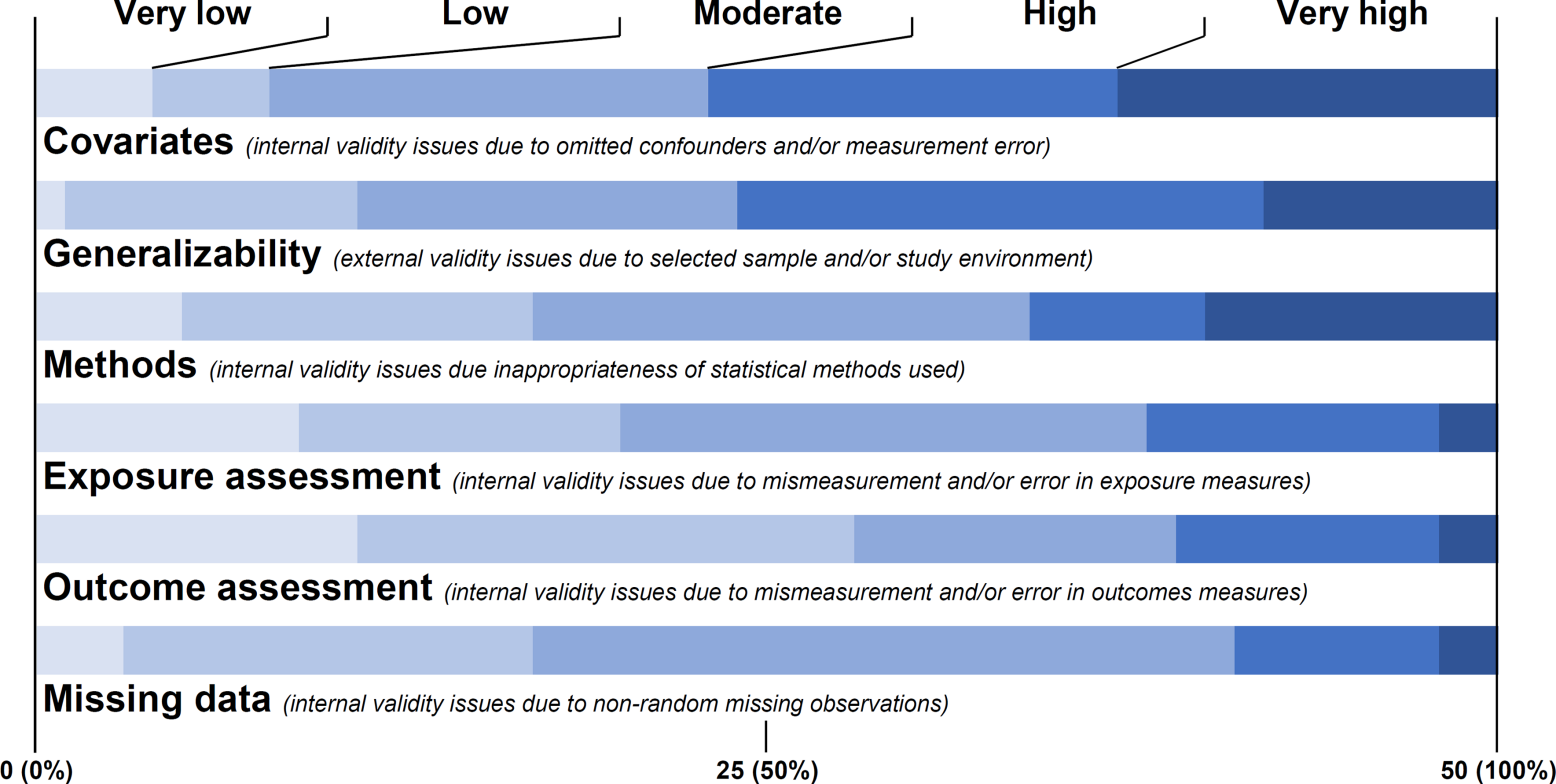
Summary of severity of issues in causal estimate in academic article by category.

Reviewers most commonly listed covariates, particularly failure to account for confounding variables, as the most severe source of bias in academic articles, listing high or very high severity of issues related to covariates for 54% (n = 27) of articles, followed by generalizability for 52% (n = 26) and statistical methods for 32% (n = 16) (Fig 2). Exposure assessment, outcome assessment, and missing data were found to be relatively minor sources of error in academic articles.

**Fig 3:**
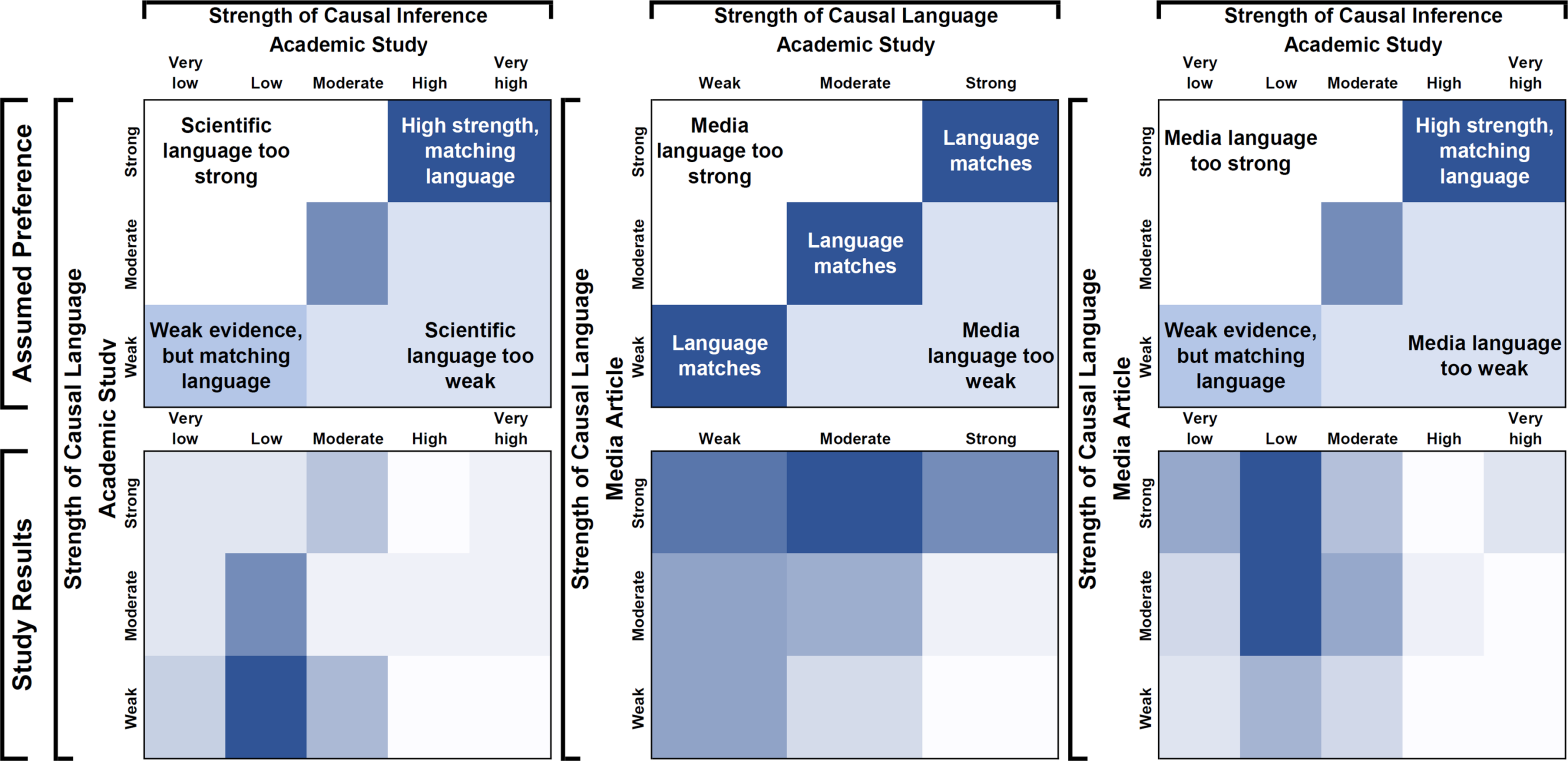
Strength of causal inference vs. strength of causal language in academic and media articles. The upper charts represent an assumed set of theoretically preferred regions, where we would prefer studies of the form exposure vs. outcome which reach the public to have language matching the strength of causal inference, a slight preference towards stronger causal inference, media language matching the academic language, and a preference for understated vs. overstated strength of causal language. The lower charts represent the empirical results of the study, where darker regions have more articles. The raw number of articles in each box is available in supplemental S4 Table.

Reviewers directly indicated that 34% (n = 17) of academic studies used causal language which was too strong for the assessed causal inference. 48% (n = 31) of media articles were written using causal language that was too strong given the language used in the academic study, which were themselves rated as being overstated on average given the strength of causal inference. As a cross-check, we further examined overstatement by comparing reviewers’ ratings of strength of causal language with their ratings of strength of causal inference. The top panels of Fig 3 show an assumed set of theoretically preferred regions where language is commensurate with strength of causal inference, and darker shades represent more preferred regions. The bottom panels show the distribution of results of our study, with darker regions indicating more articles. Using the definition in the theoretical figure regions, 42% (n = 21) of academic studies and 67% (n = 43) of media articles were found to have language which was stronger than the causal inference. There were 46 media articles linked to academic articles rated as “Low” or “Very low” strength of causal inference; the large majority (n = 38, 83%) of these studies were written about using moderate or strong causal language by the media. Among the 35 academic articles rated as “Low” or “Very low” strength of causal inference, 45% (n = 16) were written with strong or moderate causal language. More than half of the media articles (n = 35, 55%) had causal language that was stronger than the causal language used in the academic article. In 39% of cases (n = 25), the strength of causal language in the academic article matched the strength of causal language in the media article. In 6% (n = 4) media articles, the strength of causal language was weaker than the language in the academic article.

## Discussion

This study found that among the 50 health-related academic studies assessing the relationship between an exposure and outcome most shared in social media and their corresponding shared media articles, 1) only a small fraction of studies demonstrated strong causal inference between the exposure and outcome, even among those published in high-impact journals, 2) the language used in academic articles tended to slightly overstate their strength of causal inference, and 3) the media articles about them were likely to overstate strength of causal inference and inaccurately report key study characteristics and/or results. Previous studies examining overstatement of causal language utilize either language comparison and/or assumed strength of causal inference exclusively from very broad study design characteristics [12, 23, 25, 31]. This study presents two major additions to this literature: 1) a novel review tool designed to independently assess strength of causal inference, and 2) a sampling frame developed to identify the articles most likely to be consumed in social media.

The pathway to misrepresentation of evidence has components across the research production and dissemination spectrum, many of which we are unable to isolate in this study. We selected articles among those that are popularly shared in social media in order to assess the research behind popular articles that are most consumed by public audiences, and therefore this sample is unlikely to be representative of the broader work in both academia and media. While we can conclude that the majority of health research to which audiences are frequently exposed does not have strong causal inference, we cannot isolate the specific roles played by academic institutions, traditional media, social media platforms, and the public in producing this result. Review of the strength of causal inference and language in work published by authors at the top medical institutions as well as reviews of media articles across a range of popularity levels is needed to better understand where and why issues occur in the research production and dissemination pathway. While this study cannot determine whether the intent of the academic article authors was to develop strong and highly generalizable causal inference, we can examine to what degree they describe their question and/or results as such. Many of the academic article authors did not intend to make a generalizable causal argument between exposure and outcome, but in such cases our study assesses whether the language matches the strength of the causal inference.

The practical limitations of this study most likely biased our results towards finding higher strength of causal inference for the academic studies. We examined only the published article itself in the review process, and did not attempt to replicate or check calculations, data, or code, nor did we assess all possible sources of error, including cherry-picking data and results. Causal inference is weakened by any data or methodological factor unaccounted for, and any issues which were not discovered and documented by the reviewers would be more likely to weaken causal identification than strengthen it. One further practical limitation is that only 50 article sets were reviewed, though noting that these articles most likely represent over half of all social media shares in 2015.

The strength of causal inference review tool developed for this study creates a useful framework for researchers and research consumers, while also highlighting the need for additional tool development. While similar tools and frameworks have recently been proposed, most notably the ROBINS-I [44] and related Risk of Bias 2.0 [45] tools, the CLAIMS tool differs in several key respects in order to be most useful for research consumers and reviewers. Firstly, it attempts to be study design-agnostic through its frame of reference. We anchored our highest strength of causal inference on utility for decision-making by whether it approximates an often unobtainable “ideal” scenario, setting strength of causal inference as unconditional on study feasibility. Secondly, it attempts to incorporate both internal and external validity into final summary measures. Finally, it attempts to review strength of causal inference in individual studies, rather than bodies of literature.

These flexibilities come at several important costs, most notably subjectivity of resulting reviews, reduced replicability, and required resource intensity. While the former items may be mitigated in part by multiple reviews and arbitration, the latter remains a large concern. We estimate that each reviewer took 3–6 hours to complete review for each study, with three highly skilled reviewers per study. Further, while reviewers generally agreed with each other, exact agreement was far from perfect, highlighting the inherent difficulty in review of causal inference and language. Immediate improvements to the tool presented here will be focused on clarity of language among multi-disciplinary reviewers, improvements in delineations between study types and methods. Further larger scale review efforts of academic and media articles can help yield data which can be used to develop review tools with lower resource intensity requirements and greater accuracy and replicability per review, which in turn increases the feasibility and variety of potential applications.

As popular viewership and citations are tied increasingly to measuring the success of research and researchers, the pressure on scientists and journalists to select overstated, inaccurate and/or low evidence for production and/or dissemination are likely to become stronger. We speculate that the proliferation of social media and open access to research may increase ties from popular preferences to research production and selection, and that increased competition for stagnant research funding [46] is likely to exacerbate incentives to have research that stands out over that addressing areas with the highest burden of disease [47] or strength of causal inference.

Finally, we acknowledge that the results of this study are likely to be prone to misinterpretation, following popular narratives that place fault primarily on academia, media, and/or the public. In order to attempt to prevent this misinterpretation, we have provided Table 3, which lists both the main study conclusions and several potential misinterperetations and overstatements that those exposed to this study may have. We urge researchers to examine, critique and replicate this work in order to better understand both the state of causal inference and methods of examining it.

**Table 3:** Hypotheses supported and not assessed or supported by this study.

**Study finds support for these hypotheses**	
*Justification*:	*Hypotheses supported by this study*:
Primary study conclusions drawn from the main objectives, design, and results of this study. Replication, validation, and critical review of the methods and conclusions by independent parties are still necessary before results should be considered conclusive.	- The academic articles assessing the relationship between an exposure and health outcome that were most shared on social media in 2015 have, on average: - Relatively low strength of causal inference - Slightly overstated strength of causal language - The media articles that were most shared on social media in 2015 reporting on academic articles assessing the relationship between an exposure and health outcome have, on average: - Overstated strength of causal language relative to both the language used in the academic article and independently-assessed strength of causal inference in that article - Inaccurate reporting on key properties of the study.
**Study DOES NOT assess or find support for these hypotheses**	
*Justification*:	*Hypotheses not assessed/supported by this study*:
This study DOES NOT assess these hypotheses. Reporting any of these conclusions as a result of this study is inaccurate and a misrepresenting the results and conclusions of this study. At most, these hypotheses remain plausible given the results of this study, and could be considered hypothesis-generating. However, additional review studies specifically designed to assess these questions are necessary in order to add any substantial weight to these hypotheses.	- Academic institutions, including researchers, universities, and journals, produce mostly weak and/or overstated evidence. - Media institutions systematically misreport and overstate findings and/or select low strength studies on which to report. - Social media and the public systematically select and share misreported, exaggerated, and/or low strength of causal inference findings

## Supporting information

S1 Fig Summary of social media shares in sample. The x-axis shows the rank order of media articles in the combined Facebook and Twitter popularity lists by social media shares, where the first item (rank = 1) is the most popularly shared article, the second is the second most popularly shared article, etc. The y-axis shows the proportion of total shares of all URLs generated from the NewsWhip Insights search for each social media network, shown on the dark blue and red lines, respectively, where 100% is the total shares of all URLs meeting our search criteria. The lines indicate the cumulative proportion of shares reached by each rank (i.e. the proportion on the y axis at x = 3 for Facebook is the total proportion of Facebook shares reached from rank 1, 2, and 3). Number of shares is generated from sharing statistics, using the algorithm described in the Methods section to generate a popularity list order with approximately equal contribution of Facebook and Twitter. The blue area represents the 231 media articles in the combined list which were screened in order to generate the 64 media articles (and corresponding 50 academic studies) which were entered into this study. This area searched represents 68% of all Facebook shares and 45% of all Tweets of URLs meeting our search criteria.
(PNG)

S2 Fig Screening and review process summary. This diagram shows the procedure for systematically generating the review media articles and academic studies for this systematic review, as per PRISMA guidelines. Reason(s) for rejection was assessed at each level of review (media article title, article text, or academic article abstract, in that order), but were not assessed comprehensively.
(PNG)

S1 Table Domains, institutions, and journals associated with articles in sample. This table shows the URL domains, listed academic institutions, and academic journals associated with the media articles and academic studies in our sample. Each n represents one article with the associated domain/institution/journal, where the percent is out of the total # of media/academic articles, respectively. For academic institutions, the n indicates the number of studies with at least one author listed as affiliated with each institution. Affiliations were curated by authors to be aggregated at the highest level reasonable, such as at the university, hospital, or company level, so that multiple departments from the same university, for example, would count as at least one affiliation with that university.
(PDF)

S2 Table Academic articles reviewed. This table contains the citation of each academic study reviewed in our sample, and key final summary measures generated by the arbitrator of each article. Please note that the review process represents the subjective opinions of the randomly selected reviewers from our pool using an experimental review tool and process. They should be not be considered conclusive or universal rankings of any individual publication. The full comments from reviewers of each article are included in the attached full dataset. These data, as well as additional data including inter-reviewer communication, are available at metacausal.com/CLAIMS.
(PDF)

S3 Table Media articles reviewed. This table contains the authors, titles, and URL domain of each media article reviewed in our sample, the number of shares on each social media platform within a month of publication as determined by NewsWhip, and a summary measure of whether the causal language in the media article matched that of the associated academic article as generated by the arbitrator. Please note that the review process represents the subjective opinions of the randomly selected reviewers from our pool using an experimental review tool and process. They should be not be considered conclusive or universal rankings of any individual article. The full comments from reviewers of each article are included in the attached full dataset. These data, as well as additional data including inter-reviewer communication, are available at metacausal.com/CLAIMS.
(PDF)

S4 Table Number of articles in each category, by summary measures. This table corresponds to each panel in Fig 3, showing the number of articles/studies in each stratum, as determined by the arbitrating reviewers.
(PDF)

S1 File CLAIMS review tool. This file contains the full questionnaire from this study, as described in the methods section, condensed to a PDF. The full questionnaire is also available in its original form as a Google Forms survey on metacausal.com/CLAIMS.
(PDF)

S2 File Protocol brief registered with PROSPERO. This document contains the pre-registered protocol as registered with PROSPERO on August 5, 2016. The PROSPERO registration can be found at http://www.crd.york.ac.uk/PROSPERO/display_record.php?ID=CRD42016045197. The full, detailed protocol is available on metacausal.com/CLAIMS/protocol.
(PDF)

S3 File PRISMA checklist. This document contains a checklist of items as suggested by the PRISMA guidelines. The page numbers in the document refer to the pdf of the full protocol, available at metacausal.com/CLAIMS/protocol.
(PDF)

S4 File Review dataset. This dataset contains the full results from the review process, including both primary and arbitrator reviews of all articles selected into the sample of this study. Additional data, including the full results from the NewsWhip Insights search, selection screening process results, inter-reviewer communications during the review process, and manually coded / author curated data are publicly available at metacausal.com/CLAIMS.
(ZIP)
