# Supplementary figures and images for: Causal language and strength of inference in academic and media articles shared in social media (CLAIMS): A systematic review
Source: PLoS One. 2018 May 30;13(5):e0196346. doi: 10.1371/journal.pone.0196346 (PMC5976147; doi:10.1371/journal.pone.0196346)

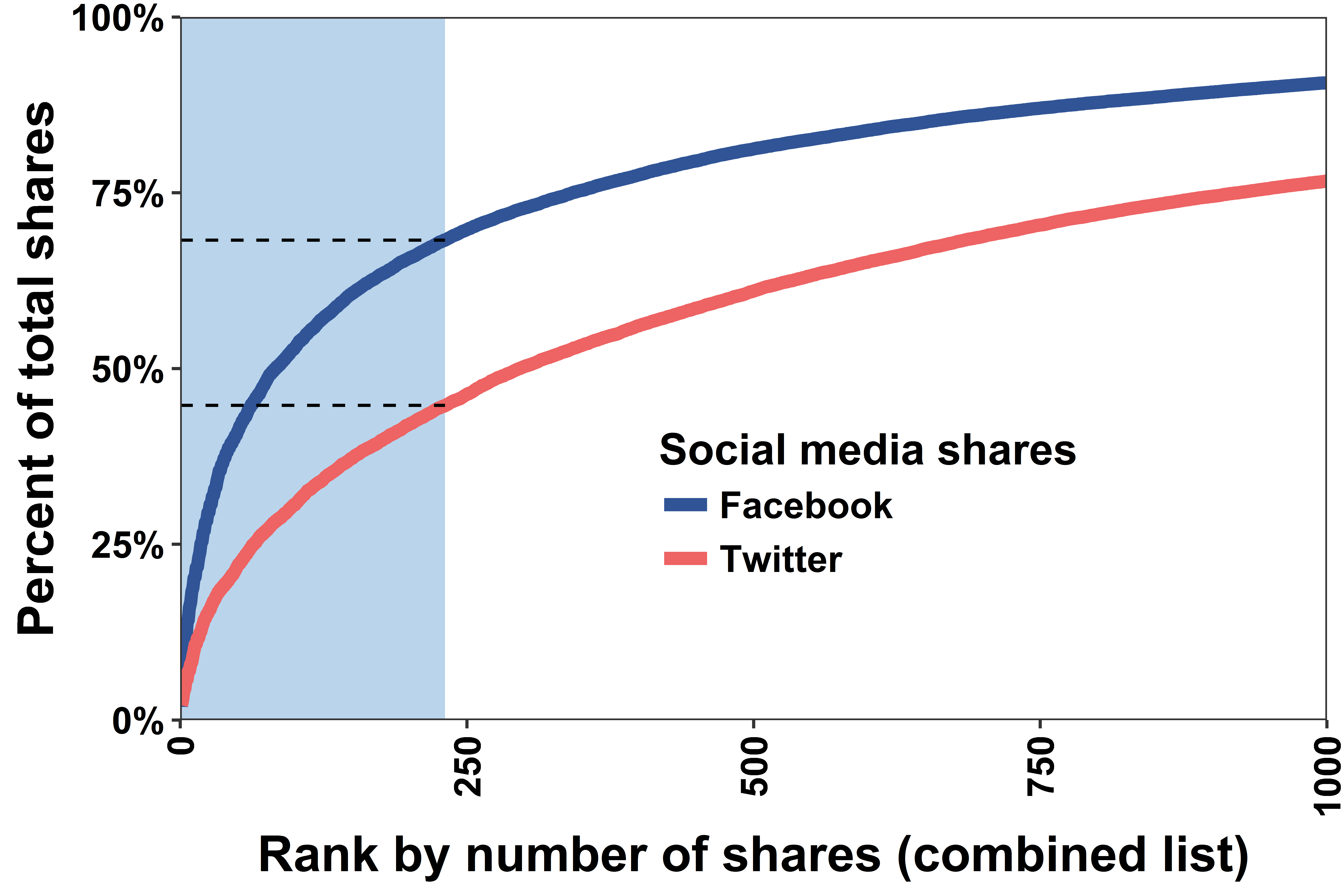

Supplement: S1 Fig — The x-axis shows the rank order of media articles in the combined Facebook and Twitter popularity lists by social media shares, where the first item (rank = 1) is the most popularly shared article, the second is the second most popularly shared article, etc. The y-axis shows the proportion of total shares of all URLs generated from the NewsWhip Insights search for each social media network, shown on the dark blue and red lines, respectively, where 100% is the total shares of all URLs meeting our search criteria. The lines indicate the cumulative proportion of shares reached by each rank (i.e. the proportion on the y axis at x = 3 for Facebook is the total proportion of Facebook shares reached from rank 1, 2, and 3). Number of shares is generated from sharing statistics, using the algorithm described in the Methods section to generate a popularity list order with approximately equal contribution of Facebook and Twitter. The blue area represents the 231 media articles in the combined list which were screened in order to generate the 64 media articles (and corresponding 50 academic studies) which were entered into this study. This area searched represents 68% of all Facebook shares and 45% of all Tweets of URLs meeting our search criteria. (PNG) [file pone.0196346.s001.png]

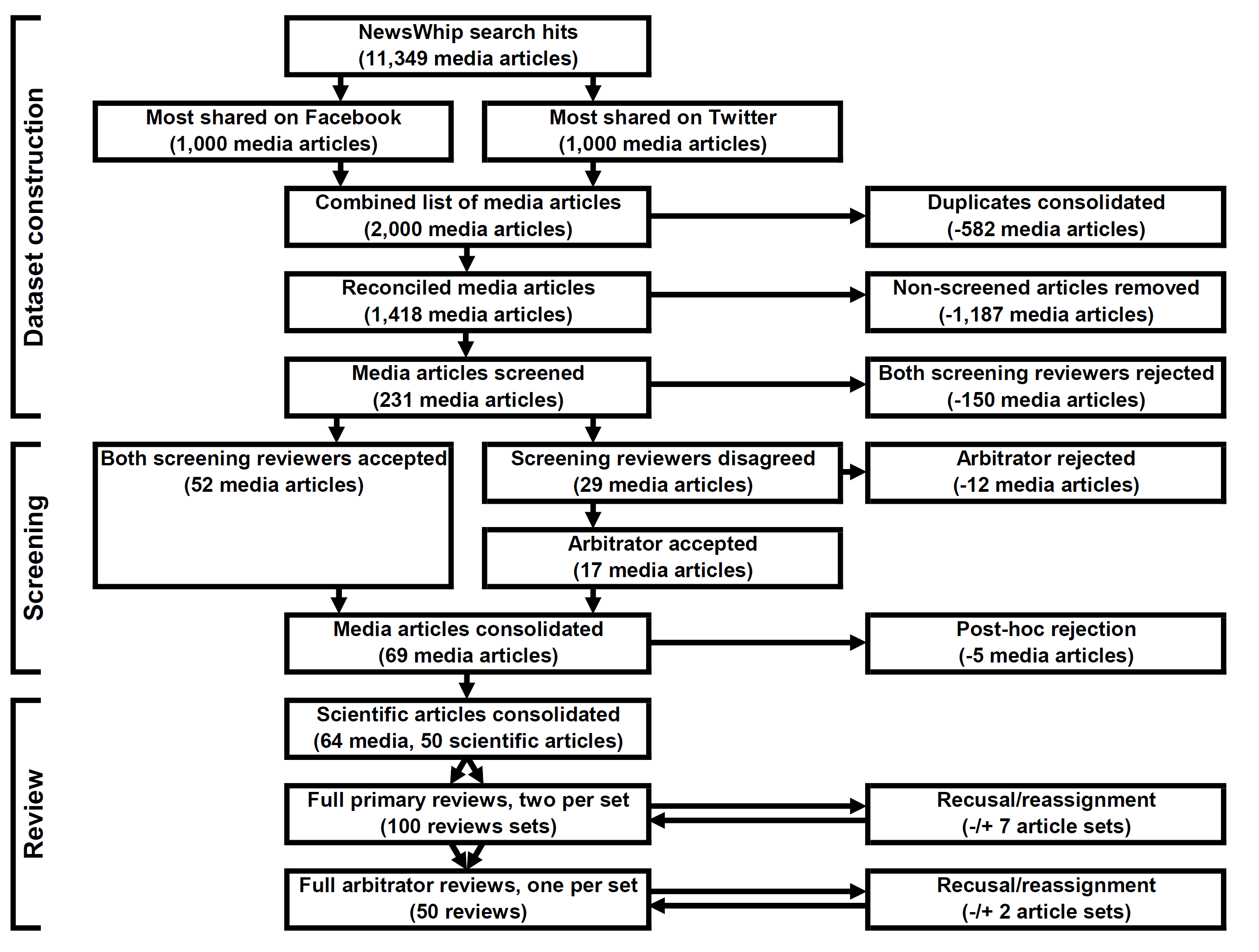

Supplement: S2 Fig — This diagram shows the procedure for systematically generating the review media articles and academic studies for this systematic review, as per PRISMA guidelines. Reason(s) for rejection was assessed at each level of review (media article title, article text, or academic article abstract, in that order), but were not assessed comprehensively. (PNG) [file pone.0196346.s002.png]
