# Supplementary material for: Causal language and strength of inference in academic and media articles shared in social media (CLAIMS): A systematic review
Source: PLoS One. 2018 May 30;13(5):e0196346. doi: 10.1371/journal.pone.0196346 (PMC5976147; doi:10.1371/journal.pone.0196346)
Supplement: S1 Table — This table shows the URL domains, listed academic institutions, and academic journals associated with the media articles and academic studies in our sample. Each n represents one article with the associated domain/institution/journal, where the percent is out of the total # of media/academic articles, respectively. For academic institutions, the n indicates the number of studies with at least one author listed as affiliated with each institution. Affiliations were curated by authors to be aggregated at the highest level reasonable, such as at the university, hospital, or company level, so that multiple departments from the same university, for example, would count as at least one affiliation with that university. (PDF) [file pone.0196346.s003.pdf]

**Media domains**	% (n)
cbsnews.com	9% (6)
nytimes.com	9% (6)
theguardian.com	9% (6)
latimes.com	6% (4)
npr.org	6% (4)
yahoo.com	6% (4)
bbc.co.uk	5% (3)
independent.co.uk	5% (3)
washingtonpost.com	5% (3)
cnn.com	3% (2)
today.com	3% (2)
aarp.org	2% (1)
bustle.com	2% (1)
citylab.com	2% (1)
dailymail.co.uk	2% (1)
globalnews.ca	2% (1)
harvard.edu	2% (1)
hellogiggles.com	2% (1)
huffingtonpost.com	2% (1)
indiatimes.com	2% (1)
instinctmagazine.com	2% (1)
news.yahoo.com	2% (1)
nydailynews.com	2% (1)
nypost.com	2% (1)
rt.com	2% (1)
sky.com	2% (1)
telegraph.co.uk	2% (1)
theautismsite.com	2% (1)
thegailygrind.com	2% (1)
thetrace.org	2% (1)
thinkprogress.org	2% (1)
yale.edu	2% (1)

Total: 32 domains
Denominator: 64 media articles

**Institutions**	% (n)
Harvard University	18% (9)
Johns Hopkins University	8% (4)
Brigham and Women's Hospital	6% (3)
Duke University	6% (3)
Indiana University	6% (3)
Massachusetts General Hospital	6% (3)
Stanford University	6% (3)
University College London	6% (3)
University of California San Francisco	6% (3)
Yale University	6% (3)
Academic Medical Center	4% (2)
CIBER of Epidemiology and Public Health	4% (2)
George Washington University	4% (2)
London School of Economics and Political Science	4% (2)
New York University	4% (2)
University of Aberdeen	4% (2)
University of California Los Angeles	4% (2)
University of São Paulo	4% (2)
University of Southern California	4% (2)
Uppsala University	4% (2)
AARP	2% (1)
Aga Khan University	2% (1)
Alliant International University	2% (1)
American Cancer Society	2% (1)
Baptist Health South Florida	2% (1)
Bassett Medical Center	2% (1)
Boston College	2% (1)
Boston University	2% (1)
Cancer Care Ontario	2% (1)
Centre for Research in Environmental Epidemiology	2% (1)
Chinese Academy of Medical Sciences	2% (1)
Chulalongkorn University	2% (1)
Clinical Center Nutrition Department	2% (1)
Columbia University	2% (1)
Dartmouth University	2% (1)
. . .	

Total: 192 institutions
Denominator: 50 academic studies

**Journals (impact factor)**	% (n)
J Epidemiol Community Health (4)	8% (4)
Am J Public Health (4)	6% (3)
PLOS One (3)	6% (3)
BMJ (17)	4% (2)
Circulation (17)	4% (2)
Heart (6)	4% (2)
Hypertension (6)	4% (2)
J Am Coll Cardiol (18)	4% (2)
Lancet (44)	4% (2)
N Engl J Med (60)	4% (2)
Proc Natl Acad Sci U S A (9)	4% (2)
Ann Behav Med (4)	2% (1)
Cell Metab (17)	2% (1)
Computers in Human Behavior (3)	2% (1)
Demography (3)	2% (1)
Environ Health Perspect (8)	2% (1)
Int J Environ Res Public Health (2)	2% (1)
J Adolesc Health (4)	2% (1)
J Am Coll Surg (4)	2% (1)
J Natl Cancer Inst (14)	2% (1)
J Neurotrauma (4)	2% (1)
J Public Health (Oxf) (2)	2% (1)
JAMA (38)	2% (1)
JAMA Intern Med (14)	2% (1)
JAMA Pediatr (10)	2% (1)
Lancet Psychiatry (6)	2% (1)
Obesity (Silver Spring) (4)	2% (1)
Pediatrics (5)	2% (1)
PLOS Genet (7)	2% (1)
PLOS Med (14)	2% (1)
Prev Chronic Dis (2)	2% (1)
Prev Med (3)	2% (1)
Psychol Aging (3)	2% (1)
Schizophr Res (4)	2% (1)
Sci Rep (5)	2% (1)

Total: 35 journals, impact factor 2-60
Denominator: 50 academic studies
