# Supplementary material for: Causal language and strength of inference in academic and media articles shared in social media (CLAIMS): A systematic review
Source: PLoS One. 2018 May 30;13(5):e0196346. doi: 10.1371/journal.pone.0196346 (PMC5976147; doi:10.1371/journal.pone.0196346)
Supplement: S2 Table — This table contains the citation of each academic study reviewed in our sample, and key final summary measures generated by the arbitrator of each article. Please note that the review process represents the subjective opinions of the randomly selected reviewers from our pool using an experimental review tool and process. They should be not be considered conclusive or universal rankings of any individual publication. The full comments from reviewers of each article are included in the attached full dataset. These data, as well as additional data including inter-reviewer communication, are available at metacausal.com/CLAIMS. (PDF) [file pone.0196346.s004.pdf]

Citation	Strength of caual inference	Most severe issue (severity)	Language matches strength
Allen, J.G., MacNaughton, P., Satish, U., Santanam, S., Vallarino, J., and Spengler, J.D., Associations of Cognitive Function Scores with Carbon Dioxide, Ventilation, and Volatile Organic Compound Exposures in Office Workers: A Controlled Exposure Study of Green and Conventional Office Environments. Environ Health Perspect, 2016. 124(6): p. 805-12	Very high	Generalizability (Low)	No, language too weak for strength of causal inference
Andersson, O., Lindquist, B., Lindgren, M., Stjernqvist, K., Domellof, M., and Hellstrom-Westas, L., Effect of Delayed Cord Clamping on Neurodevelopment at 4 Years of Age: A Randomized Clinical Trial. JAMA Pediatr, 2015. 169(7): p. 631-8	Moderate	Generalizability (High)	Yes, language matches strength of causal inference
Barr, B., Taylor-Robinson, D., Stuckler, D., Loopstra, R., Reeves, A., and Whitehead, M., 'First, do no harm': are disability assessments associated with adverse trends in mental health? A longitudinal ecological study. J Epidemiol Community Health, 2016. 70(4): p. 339-45	Moderate	Methods (Moderate)	No, language too weak for strength of causal inference
Berkman, L.F., Zheng, Y., Glymour, M.M., Avendano, M., Borsch-Supan, A., and Sabbath, E.L., Mothering alone: cross-national comparisons of later-life disability and health among women who were single mothers. J Epidemiol Community Health, 2015. 69(9): p. 865-72	Very low	Covariates, Methods (Very high)	No, language too strong for strength of causal inference
Black, M., Bhattacharya, S., Philip, S., Norman, J.E., and McLernon, D.J., Planned Cesarean Delivery at Term and Adverse Outcomes in Childhood Health. JAMA, 2015. 314(21): p. 2271-9	Low	Methods (Very high)	Yes, language matches strength of causal inference
Bratman, G.N., Hamilton, J.P., Hahn, K.S., Daily, G.C., and Gross, J.J., Nature experience reduces rumination and subgenual prefrontal cortex activation. Proc Natl Acad Sci U S A, 2015. 112(28): p. 8567-72	Moderate	Covariates, Outcome assessment (Moderate)	Yes, language matches strength of causal inference
Choi, Y., Chang, Y., Ryu, S., Cho, J., Rampal, S., Zhang, Y., Ahn, J., Lima, J.A., Shin, H., and Guallar, E., Coffee consumption and coronary artery calcium in young and middle-aged asymptomatic adults. Heart, 2015. 101(9): p. 686-91	Low	Covariates, Methods, Generalizability, Exposure assessment, Missing data (Moderate)	Yes, language matches strength of causal inference
Dadvand, P., Nieuwenhuijsen, M.J., Esnaola, M., Forns, J., Basagana, X., Alvarez-Pedrerol, M., Rivas, I., Lopez-Vicente, M., De Castro Pascual, M., Su, J., Jerrett, M., Querol, X., and Sunyer, J., Green spaces and cognitive development in primary schoolchildren. Proc Natl Acad Sci U S A, 2015. 112(26): p. 7937-42	Moderate	Outcome assessment (High)	Yes, language matches strength of causal inference
Derraik, J.G., Ahlsson, F., Lundgren, M., Jonsson, B., and Cutfield, W.S., First-borns have greater BMI and are more likely to be overweight or obese: a study of sibling pairs among 26,812 Swedish women. J Epidemiol Community Health, 2016. 70(1): p. 78-81	Low	Covariates (Very high)	Yes, language matches strength of causal inference
Ding, M., Satija, A., Bhupathiraju, S.N., Hu, Y., Sun, Q., Han, J., Lopez-Garcia, E., Willett, W., van Dam, R.M., and Hu, F.B., Association of Coffee Consumption With Total and Cause-Specific Mortality in 3 Large Prospective Cohorts. Circulation, 2015. 132(24): p. 2305-15	Moderate	Generalizability (High)	Yes, language matches strength of causal inference
Figueiredo, J.C., Hsu, L., Hutter, C.M., Lin, Y., Campbell, P.T., Baron, J.A., Berndt, S.I., Jiao, S., Casey, G., Fortini, B., Chan, A.T., Cotterchio, M., Lemire, M., Gallinger, S., Harrison, T.A., Le Marchand, L., Newcomb, P.A., Slattery, M.L., Caan, B.J., Carlson, C.S., Zanke, B.W., Rosse, S.A., Brenner, H., Giovannucci, E.L., Wu, K., Chang-Claude, J., Chanock, S.J., Curtis, K.R., Duggan, D., Gong, J., Haile, R.W., Hayes, R.B., Hoffmeister, M., Hopper, J.L., Jenkins, M.A., Kolonel, L.N., Qu, C., Rudolph, A., Schoen, R.E., Schumacher, F.R., Seminara, D., Stelling, D.L., Thibodeau, S.N., Thornquist, M., Warnick, G.S., Henderson, B.E., Ulrich, C.M., Gauderman, W.J., Potter, J.D., White, E., Peters, U., Ccfr, and Gecco, Genome-wide diet-gene interaction analyses for risk of colorectal cancer. PLoS Genet, 2014. 10(4): p. e1004228	Low	Covariates, Methods (Very high)	Yes, language matches strength of causal inference
Fountain, C., Zhang, Y., Kissin, D.M., Schieve, L.A., Jamieson, D.J., Rice, C., and Bearman, P., Association between assisted reproductive technology conception and autism in California, 1997-2007. Am J Public Health, 2015. 105(5): p. 963-71	Low	Covariates (Very high)	Yes, language matches strength of causal inference
Freemantle, N., Ray, D., McNulty, D., Rosser, D., Bennett, S., Keogh, B.E., and Pagano, D., Increased mortality associated with weekend hospital admission: a case for expanded seven day services? Bmj, 2015. 351: p. h4596	Low	Covariates, Methods (Moderate)	No, language too strong for strength of causal inference
Gadomski, A.M., Scribani, M.B., Krupa, N., Jenkins, P., Nagykaldi, Z., and Olson, A.L., Pet Dogs and Children's Health: Opportunities for Chronic Disease Prevention? Prev Chronic Dis, 2015. 12: p. E205	Very low	Covariates, Generalizability (High)	Yes, language matches strength of causal inference
Gauderman, W.J., Urman, R., Avol, E., Berhane, K., McConnell, R., Rappaport, E., Chang, R., Lurmann, F., and Gilliland, F., Association of improved air quality with lung development in children. N Engl J Med, 2015. 372(10): p. 905-13	Moderate	Covariates, Exposure assessment (Moderate)	Yes, language matches strength of causal inference
Gorman, B.K., Denney, J.T., Dowdy, H., and Medeiros, R.A., A New Piece of the Puzzle: Sexual Orientation, Gender, and Physical Health Status. Demography, 2015. 52(4): p. 1357-82	Moderate	Covariates, Generalizability, Exposure assessment, Outcome assessment, Missing data (Moderate)	Yes, language matches strength of causal inference
Hall, K.D., Bemis, T., Brychta, R., Chen, K.Y., Courville, A., Crayner, E.J., Goodwin, S., Guo, J., Howard, L., Knuth, N.D., Miller, B.V., 3rd, Prado, C.M., Siervo, M., Skarulis, M.C., Walter, M., Walter, P.J., and Yannai, L., Calorie for Calorie, Dietary Fat Restriction Results in More Body Fat Loss than Carbohydrate Restriction in People with Obesity. Cell Metab, 2015. 22(3): p. 427-36	Moderate	Generalizability (Very high)	No, language too strong for strength of causal inference

Heida, K.Y., Franx, A., van Rijn, B.B., Eijkemans, M.J., Boer, J.M., Verschuren, M.W., Oudijk, M.A., Bots, M.L., and van der Schouw, Y.T., Earlier Age of Onset of Chronic Hypertension and Type 2 Diabetes Mellitus After a Hypertensive Disorder of Pregnancy or Gestational Diabetes Mellitus. Hypertension, 2015. 66(6): p. 1116-22	Low	Covariates (Very high)	No, language too strong for strength of causal inference
Hemenway, D. and Solnick, S.J., The epidemiology of self-defense gun use: evidence from the National Crime Victimization Surveys 2007-2011. Prev Med, 2015. 79: p. 22-7	Low	Covariates, Methods, Exposure assessment, Outcome assessment, Missing data (Very high)	Yes, language matches strength of causal inference
Insight Start Study Group, Lundgren, J.D., Babiker, A.G., Gordin, F., Emery, S., Grund, B., Sharma, S., Avihingsanon, A., Cooper, D.A., Fatkenheuer, G., Llibre, J.M., Molina, J.M., Munderi, P., Schechter, M., Wood, R., Klingman, K.L., Collins, S., Lane, H.C., Phillips, A.N., and Neaton, J.D., Initiation of Antiretroviral Therapy in Early Asymptomatic HIV Infection. N Engl J Med, 2015. 373(9): p. 795-807	Very high	Covariates, Methods, Generalizability, Outcome assessment, Missing data (Low)	Yes, language matches strength of causal inference
Jones, D.E., Greenberg, M., and Crowley, M., Early Social-Emotional Functioning and Public Health: The Relationship Between Kindergarten Social Competence and Future Wellness. Am J Public Health, 2015. 105(11): p. 2283-90	Low	Exposure assessment (Very high)	Yes, language matches strength of causal inference
Kardan, O., Gozdyra, P., Misic, B., Moola, F., Palmer, L.J., Paus, T., and Berman, M.G., Neighborhood greenspace and health in a large urban center. Sci Rep, 2015. 5: p. 11610	Low	Methods (Very high)	No, language too strong for strength of causal inference
Kovesdy, C.P., Norris, K.C., Boulware, L.E., Lu, J.L., Ma, J.Z., Streja, E., Molnar, M.Z., and Kalantar-Zadeh, K., Association of Race With Mortality and Cardiovascular Events in a Large Cohort of US Veterans. Circulation, 2015. 132(16): p. 1538-48	Low	Generalizability (Very high)	No, language too strong for strength of causal inference
Kwok, C.S., Boekholdt, S.M., Lentjes, M.A., Loke, Y.K., Luben, R.N., Yeong, J.K., Wareham, N.J., Myint, P.K., and Khaw, K.T., Habitual chocolate consumption and risk of cardiovascular disease among healthy men and women. Heart, 2015. 101(16): p. 1279-87	Low	Covariates, Methods, Generalizability, Exposure assessment (High)	Yes, language matches strength of causal inference
Leong, D.P., Teo, K.K., Rangarajan, S., Lopez-Jaramillo, P., Avezum, A., Jr., Orlandini, A., Seron, P., Ahmed, S.H., Rosengren, A., Kelishadi, R., Rahman, O., Swaminathan, S., Iqbal, R., Gupta, R., Lear, S.A., Oguz, A., Yusoff, K., Zatonska, K., Chifamba, J., Igumbor, E., Mohan, V., Anjana, R.M., Gu, H., Li, W., and Yusuf, S., Prognostic value of grip strength: findings from the Prospective Urban Rural Epidemiology (PURE) study. Lancet, 2015. 386(9990): p. 266-73	Very low	Covariates, Exposure assessment (Moderate)	Yes, language matches strength of causal inference
Lereya, S.T., Copeland, W.E., Costello, E.J., and Wolke, D., Adult mental health consequences of peer bullying and maltreatment in childhood: two cohorts in two countries. Lancet Psychiatry, 2015. 2(6): p. 524-31	Very low	Covariates, Missing data (Very high)	No, language too strong for strength of causal inference
Levy, B.R., Ferrucci, L., Zonderman, A.B., Slade, M.D., Troncoso, J., and Resnick, S.M., A culture-brain link: Negative age stereotypes predict Alzheimer's disease biomarkers. Psychol Aging, 2016. 31(1): p. 82-8	Low	Covariates, Generalizability (High)	No, language too strong for strength of causal inference
Liu, A.Y., Cohen, S.E., Vittinghoff, E., Anderson, P.L., Doblecki-Lewis, S., Bacon, O., Chege, W., Postle, B.S., Matheson, T., Amico, K.R., Liegler, T., Rawlings, M.K., Trainor, N., Blue, R.W., Estrada, Y., Coleman, M.E., Cardenas, G., Feaster, D.J., Grant, R., Philip, S.S., Elion, R., Buchbinder, S., and Kolber, M.A., Preexposure Prophylaxis for HIV Infection Integrated With Municipal- and Community-Based Sexual Health Services. JAMA Intern Med, 2016. 176(1): p. 75-84	Low	Generalizability (Very high)	No, language too strong for strength of causal inference
Liu, B., Floud, S., Pirie, K., Green, J., Peto, R., and Beral, V., Does happiness itself directly affect mortality? The prospective UK Million Women Study. Lancet, 2016. 387(10021): p. 874-81	Moderate	Exposure assessment (High)	No, language too strong for strength of causal inference
Loftfield, E., Freedman, N.D., Graubard, B.I., Hollenbeck, A.R., Shebl, F.M., Mayne, S.T., and Sinha, R., Coffee drinking and cutaneous melanoma risk in the NIH-AARP diet and health study. J Natl Cancer Inst, 2015. 107(2)	Low	Outcome assessment (Very high)	Yes, language matches strength of causal inference
Lopez, D.S., Wang, R., Tsilidis, K.K., Zhu, H., Daniel, C.R., Sinha, A., and Canfield, S., Role of Caffeine Intake on Erectile Dysfunction in US Men: Results from NHANES 2001-2004. PLoS One, 2014. 10(4): p. e0123547	Very low	Covariates, Generalizability, Exposure assessment, Outcome assessment, Missing data (High)	Yes, language matches strength of causal inference
Lustig, R.H., Mulligan, K., Noworolski, S.M., Tai, V.W., Wen, M.J., Erkin-Cakmak, A., Gugliucci, A., and Schwarz, J.M., Isocaloric fructose restriction and metabolic improvement in children with obesity and metabolic syndrome. Obesity (Silver Spring), 2016. 24(2): p. 453-60	Low	Generalizability (High)	Yes, language matches strength of causal inference
Melis, G., Gelormino, E., Marra, G., Ferracin, E., and Costa, G., The Effects of the Urban Built Environment on Mental Health: A Cohort Study in a Large Northern Italian City. Int J Environ Res Public Health, 2015. 12(11): p. 14898-915	Very low	Covariates, Generalizability (Very high)	No, language too strong for strength of causal inference
Mokry, L.E., Ross, S., Ahmad, O.S., Forgetta, V., Smith, G.D., Goltzman, D., Leong, A., Greenwood, C.M., Thanassoulis, G., and Richards, J.B., Vitamin D and Risk of Multiple Sclerosis: A Mendelian Randomization Study. PLoS Med, 2015. 12(8): p. e1001866	High	Generalizability (Moderate)	Yes, language matches strength of causal inference
Myrick, J.G., Emotion regulation, procrastination, and watching cat videos online: Who watches Internet cats, why, and to what effect? Computers in Human Behavior, 2015. 52: p. 168-176	Low	Generalizability, Outcome assessment, Missing data (High)	Yes, language matches strength of causal inference

Nasir, K., Bittencourt, M.S., Blaha, M.J., Blankstein, R., Agatson, A.S., Rivera, J.J., Miedema, M.D., Sibley, C.T., Shaw, L.J., Blumenthal, R.S., Budoff, M.J., and Krumholz, H.M., Implications of Coronary Artery Calcium Testing Among Statin Candidates According to American College of Cardiology/American Heart Association Cholesterol Management Guidelines: MESA (Multi-Ethnic Study of Atherosclerosis). J Am Coll Cardiol,	Moderate	Exposure assessment (Moderate)	Yes, language matches strength of causal inference
Nemeth, B., Scheres, L.J., Lijfering, W.M., and Rosendaal, F.R., Bloodcurdling movies and measures of coagulation: Fear Factor crossover trial. BMJ, 2015. 351: p. h6367	Low	Covariates, Methods, Generalizability (Very high)	Yes, language matches strength of causal inference
Peckham, S., Lowery, D., and Spencer, S., Are fluoride levels in drinking water associated with hypothyroidism prevalence in England? A large observational study of GP practice data and fluoride levels in drinking water. J Epidemiol Community Health, 2015. 69(7): p. 619-24	Low	Covariates, Methods, Exposure assessment (High)	No, language too strong for strength of causal inference
Ploubidis, G.B., Silverwood, R.J., DeStavola, B., and Grundy, E., Life-Course Partnership Status and Biomarkers in Midlife: Evidence From the 1958 British Birth Cohort. Am J Public Health, 2015. 105(8): p. 1596-603	Low	Generalizability (Very high)	Yes, language matches strength of causal inference
Quach, D., Jastrowski Mano, K.E., and Alexander, K., A Randomized Controlled Trial Examining the Effect of Mindfulness Meditation on Working Memory Capacity in Adolescents. J Adolesc Health, 2016. 58(5): p. 489-96	Moderate	Generalizability (Very high)	No, language too strong for strength of causal inference
Salles, A., Mueller, C.M., and Cohen, G.L., Exploring the Relationship Between Stereotype Perception and Residents' Well-Being. J Am Coll Surg, 2016. 222(1): p. 52-8	Low	Methods (Very high)	Yes, language matches strength of causal inference
Shah, N.H., LePendu, P., Bauer-Mehren, A., Ghebremariam, Y.T., Iyer, S.V., Marcus, J., Nead, K.T., Cooke, J.P., and Leeper, N.J., Proton Pump Inhibitor Usage and the Risk of Myocardial Infarction in the General Population. PLoS One, 2015. 10(6): p. e0124653	Low	Covariates, Exposure assessment (High)	No, language too strong for strength of causal inference
Stahl, J.E., Dossett, M.L., LaJoie, A.S., Denninger, J.W., Mehta, D.H., Goldman, R., Fricchione, G.L., and Benson, H., Relaxation Response and Resiliency Training and Its Effect on Healthcare Resource Utilization. PLoS One, 2015. 10(10): p. e0140212	Low	Covariates (High)	No, language too strong for strength of causal inference
Stamm, J.M., Koerte, I.K., Muehlmann, M., Pasternak, O., Bourlas, A.P., Baugh, C.M., Giwerc, M.Y., Zhu, A., Coleman, M.J., Bouix, S., Fritts, N.G., Martin, B.M., Chaisson, C., McClean, M.D., Lin, A.P., Cantu, R.C., Tripodis, Y., Stern, R.A., and Shenton, M.E., Age at First Exposure to Football Is Associated with Altered Corpus Callosum White Matter Microstructure in Former Professional Football Players. J Neurotrauma, 2015. 32(22): p. 1768-76	Low	Covariates, Generalizability (High)	Yes, language matches strength of causal inference
Torrey, E.F., Simmons, W., and Yolken, R.H., Is childhood cat ownership a risk factor for schizophrenia later in life? Schizophr Res, 2015. 165(1): p. 1-2	Low	Methods (Very high)	Yes, language matches strength of causal inference
Trasande, L. and Attina, T.M., Association of exposure to di-2-ethylhexylphthalate replacements with increased blood pressure in children and adolescents. Hypertension, 2015. 66(2): p. 301-8	Low	Covariates, Methods (Very high)	Yes, language matches strength of causal inference
Winning, A., Glymour, M.M., McCormick, M.C., Gilsanz, P., and Kubzansky, L.D., Psychological Distress Across the Life Course and Cardiometabolic Risk: Findings From the 1958 British Birth Cohort Study. J Am Coll Cardiol, 2015. 66(14): p. 1577-86	Moderate	Generalizability (High)	Yes, language matches strength of causal inference
Wood, C.J., Pretty, J., and Griffin, M., A case-control study of the health and well-being benefits of allotment gardening. J Public Health (Oxf), 2015	Low	Covariates (Very high)	No, language too strong for strength of causal inference
Zawadzki, M.J., Smyth, J.M., and Costigan, H.J., Real-Time Associations Between Engaging in Leisure and Daily Health and Well-Being. Ann Behav Med, 2015. 49(4): p. 605-15	Very low	Covariates, Methods, Generalizability (Very high)	No, language too strong for strength of causal inference
Zucker, N., Copeland, W., Franz, L., Carpenter, K., Keeling, L., Angold, A., and Egger, H., Psychological and Psychosocial Impairment in Preschoolers With Selective Eating. Pediatrics, 2015. 136(3): p. e582-90	Very low	Covariates (Very high)	Yes, language matches strength of causal inference
