# Supplementary material for: Causal language and strength of inference in academic and media articles shared in social media (CLAIMS): A systematic review
Source: PLoS One. 2018 May 30;13(5):e0196346. doi: 10.1371/journal.pone.0196346 (PMC5976147; doi:10.1371/journal.pone.0196346)
Supplement: S3 Table — This table contains the authors, titles, and URL domain of each media article reviewed in our sample, the number of shares on each social media platform within a month of publication as determined by NewsWhip, and a summary measure of whether the causal language in the media article matched that of the associated academic article as generated by the arbitrator. Please note that the review process represents the subjective opinions of the randomly selected reviewers from our pool using an experimental review tool and process. They should be not be considered conclusive or universal rankings of any individual article. The full comments from reviewers of each article are included in the attached full dataset. These data, as well as additional data including inter-reviewer communication, are available at metacausal.com/CLAIMS. (PDF) [file pone.0196346.s005.pdf]

Author(s)	Headline	Domain	Facebook shares	Twitter shares	Language matches scientific article
Chris Mooney	Scientists have discovered that living near trees is good for your health	washingtonpost.com	61,771	2,919	Yes, language matches
Amy Kraft	Cat parasite Toxoplasma gondii linked to mental illness, schizophrenia	cbsnews.com	38,125	1,663	No, language stronger in media
Anahad O'Connor	Cutting Sugar Improves Children's Health in Just 10 Days	nytimes.com	18,949	3,245	Yes, language matches
Denise Grady	Happiness Doesn’t Bring Good Health, Study Finds	nytimes.com	4,373	2,790	Yes, language matches
Allison Aubrey	Drink To Your Health: Study Links Daily Coffee Habit To Longevity	npr.org	17,260	1,904	Yes, language matches
Not listed	Jeremy Hunt 'misrepresented weekend deaths data'	bbc.co.uk	3,512	2,458	No, language stronger in media
Joe Romm	Exclusive: Landmark Study Finds Elevated Carbon Dioxide Levels Impair Human Brain Function	thinkprogress.org	2,917	2,206	No, language stronger in media
Sanjay Gupta	Cutting back sugar improves kids' health in 10 days	cnn.com	13,264	918	No, language stronger in media
Tara Haelle	Delayed Umbilical Cord Clamping May Benefit Children Years Later	npr.org	12,642	847	Yes, language matches
Sue McGreevey	Relaxation response proves positive	harvard.edu	3,909	1,626	Yes, language matches
Charlie Cooper	Has a new academic study revealed the true cost of Iain Duncan Smith's welfare reforms	independent.co.uk	1,939	1,563	Yes, language matches
Alan Zarembo	Black patients fare better than whites when both get same healthcare, study finds	latimes.com	420	1,495	No, language stronger in media
Evan Deflippis, Devin Hughes	New Study Finds No Advantages to Defensive Gun Use	thetrace.org	8,161	578	No, language stronger in media
Donald G. McNeil, Jr	H.I.V. Treatment Should Start With Diagnosis, U.S. Health Officials Say	nytimes.com	1,915	1,463	Yes, language matches
Deborah Netburn	Science proves what you suspected: hiking's good for your mental health	latimes.com	6,588	1,107	No, language stronger in media
Allison Aubrey	Childhood Stress May Prime Pump For Chronic Disease Later	npr.org	6,363	1,141	Yes, language matches
Amy Kraft	New research on heart health benefits of chocolate	cbsnews.com	6,282	499	No, language stronger in media
Ariana Eunjung Cha	Good news for chocolate lovers: Study suggests the more you eat, the lower your risk of heart disease	washingtonpost.com	5,568	198	No, language stronger in media
James Gallagher	Low-fat diets 'better than cutting carbs' for weight loss	bbc.co.uk	2,494	1,303	Yes, language matches
Not listed	Drinking Coffee Gives You A Stronger Penis According To New Study	instinctmagazine.com	5,411	177	No, language stronger in media
Jan Hoffman	Children's Lung Health Improves as Air Pollution Is Reduced, Study Says	nytimes.com	981	1,143	No, language stronger in media
Kelly Wallace	Kindergartners' behavior tied to adult success	cnn.com	4,419	603	No, language stronger in media
Peter Walker	Horror health warning? Scary films really can curdle the blood	theguardian.com	371	1,040	No, language stronger in media
James Gallagher	Palm 'holds secrets of future health'	bbc.co.uk	1,016	939	Yes, language matches
Carolyn Gregoire	How Green Spaces In Schools Can Optimize Students' Learning And Productivity	huffingtonpost.com	3,699	366	No, language stronger in media
Karen Kaplan	Bullying does more long-term mental health harm than abuse, study says	latimes.com	1,917	937	Yes, language matches
Sarah Boseley	Health threat of sugar is vastly underestimated, study	theguardian.com	2,602	908	Yes, language matches
Ashley Welch	Coffee drinking linked to a longer life	cbsnews.com	3,570	637	No, language stronger in media
Andrew M. Seaman	Pill protects against HIV in real-world settings	yahoo.com	34	889	No, language stronger in media
Candy Sagon	Some Acid Reflux Medication Could Increase Heart Attack Risk	aarp.org	3,280	66	Yes, language matches
Not listed	Coffee-drinkers less likely to die from certain diseases	yahoo.com	2,117	824	Yes, language matches
Linda Carroll	'No head trauma is good head trauma': Kids' sports under scrutiny	today.com	3,198	142	No, language stronger in media
Roni Rabin	C-Sections Are Best With a Little Labor, a Study Says	nytimes.com	2,321	820	No, language stronger in media
Jill Layton	Research says, having a dog as a kid is amazing for your mental health	hellogiggles.com	3,173	30	No, language stronger in media
Ben Spencer	Why firstborn girls are more likely to get fat	dailymail.co.uk	2,876	72	Yes, language matches
Michael Greenwood	Negative beliefs about aging predict Alzheimer’s disease in Yale-led study	yale.edu	545	740	Yes, language matches
Not listed	Coffee Prevents Heart Attacks, Study Says	sky.com	2,825	330	No, language stronger in media
Ian Johnston	Angry, stressed or depressed? Gardening can help, new study finds	independent.co.uk	1,463	724	Yes, language matches
Sarah Boseley	Bullied children have 'greater mental health risks' than those maltreated by adults	theguardian.com	749	721	Yes, language matches
Linda Carroll	'Drip, drip, drip' of daily stress: Single parenting takes health toll	today.com	2,780	55	No, language stronger in media
Paige Turner	How You Get Pregnant Has a Correlation to Your Child’s Risk of Autism	theautismsite.com	2,747	19	No, language weaker in media
Ashley Welch	Picky eating in kids could be sign of bigger health concerns	cbsnews.com	2,718	200	Yes, language matches
Monica Tan	More trees on your street means fewer health problems, says study	theguardian.com	2,699	514	Yes, language matches
Samantha Bonar	Coffee consumption linked to lower melanoma risk	latimes.com	2,544	460	No, language stronger in media
Eric Jaffe	Why Having Access to Public Transit Might Be Good for Your Mental Health	citylab.com	939	670	Yes, language matches

Meredith Engel	Microwaving food in plastic linked to diabetes: study	nydailynews.com	2,311	82	No, language stronger in media
Gina Kolata	Heart Scan Can Fine-Tune Risk Estimate for Patients Considering Statins	nytimes.com	355	659	Yes, language matches
Adrian Garcia	Study Finds Drinking Lots Of Coffee Makes Your Penis Stronger	thegailygrind.com	2,141	73	No, language stronger in media
Sarah Knapton	Marriage is more beneficial for men than women, study shows	telegraph.co.uk	1,186	640	No, language stronger in media
Not listed	Coffee may reduce risk for malignant melanoma	cbsnews.com	1,397	639	No, language stronger in media
Amy Kraft	Cat videos may be good for your health	cbsnews.com	2,083	313	Yes, language matches
Kathryn Doyle	Mindfulness meditation may improve memory for teens	yahoo.com	11	610	No, language weaker in media
Rose Troup Buchanan	What happens to your body after you give up sugar	indiatimes.com	2,072	100	Yes, language matches
Kashmira Gander	How watching cat videos is good for you	independent.co.uk	384	603	Yes, language matches
Jada Green	Want a stronger penis? Drink more coffee	nypost.com	1,993	148	No, language stronger in media
Lisa Rapaport	Hypertension in pregnancy linked to future heart disease	yahoo.com	4	597	No, language weaker in media
Peter Whoriskey	How risky are bacon and hot dogs? Depends on your DNA.	washingtonpost.com	152	566	No, language stronger in media
Karen McVeigh	Fit-for-work tests may have taken serious toll on mental health – study	theguardian.com	765	560	Yes, language matches
Not listed	Lack of vitamin D may cause multiple sclerosis, study finds	theguardian.com	1,879	218	Yes, language matches
Tania Kohut	Children with pet dogs have less anxiety: study	globalnews.ca	1,853	20	No, language stronger in media
Patti Neighmond	Mellow Pastimes Can Be Good For Your Health, Too	npr.org	12	557	No, language stronger in media
Sergio Perez	‘Fluoride in water could cause depression, weight gain’ – scientists	rt.com	382	543	Yes, language matches
Laken Howard	Bisexuals Report Poorer Health Than Gay And Straight Peers, New Study Finds, Plus 3 More Reasons We Need to Be More Accepting	bustle.com	1,639	8	Yes, language matches
Kathryn Doyle	Stereotyping makes life harder for young female surgeons	news.yahoo.com	3	523	No, language stronger in media
