# Supplementary material for: Causal language and strength of inference in academic and media articles shared in social media (CLAIMS): A systematic review
Source: PLoS One. 2018 May 30;13(5):e0196346. doi: 10.1371/journal.pone.0196346 (PMC5976147; doi:10.1371/journal.pone.0196346)
Supplement: S4 Table — This table corresponds to each panel in Fig 3, showing the number of articles/studies in each stratum, as determined by the arbitrating reviewers. (PDF) [file pone.0196346.s006.pdf]

Study Results

Strength of Causal Academic Study	Strength of Causal Inference Academic Study: Very low	Low	Moderate	High	Very high
Strong	2	2	5	0	1
Moderate	2	10	1	1	1
Weak	4	15	6	0	0

Strength of Causal Media Article	Strength of Causal Language Academic Study: Weak	Moderate	Strong
Strong	12	15	10
Moderate	8	7	1
Weak	8	3	0

Strength of Causal Media Article	Strength of Causal Inference Academic Study: Very low	Low	Moderate	High	Very high
Strong	7	14	5	0	2
Moderate	3	14	7	1	0
Weak	2	6	3	0	0
