# Supplementary material for: Causal language and strength of inference in academic and media articles shared in social media (CLAIMS): A systematic review
Source: PLoS One. 2018 May 30;13(5):e0196346. doi: 10.1371/journal.pone.0196346 (PMC5976147; doi:10.1371/journal.pone.0196346)
Supplement: S1 File — This file contains the full questionnaire from this study, as described in the methods section, condensed to a PDF. The full questionnaire is also available in its original form as a Google Forms survey on metacausal.com/CLAIMS. (PDF) [file pone.0196346.s007.pdf]

CLAIMS Extraction Form v1.2

Section A. Reviewer and study information

A1. Reviewer ID

A2. Scientific study ID

A3a. Media article ID #1

A3b. Media article ID #2 (if applicable)

A3c. Media article ID #3 (if applicable)

A3d. Media article ID #4 (if applicable)

A4. Do you agree to review this study?
You have the option of recusing yourself from this study if you believe you are insufficiently equipped to give unbiased, fair, and thorough review of the assigned scientific and media article(s). You must recuse yourself from reviewing for any of the following reasons: professional/personal relationship with the main study authors, any financial conflict of interest, any social conflict of interest, lack of sufficient expertise in the methods used in the scientific articles, sufficient lack of content area knowledge, or any other perceived influence on your ability to review the assigned articles.

- Yes. I am sufficiently equipped to give an unbiased, fair, and thorough review of the assigned scientific and media articles, as outlined above.
- No. I choose to recuse myself from reviewing this study.

A4a. If "no", please state why.

Section B: Study overview

B1. List the associated Institutions/Industries (Author information + COI)

B1a. What type(s) of institution are associated?

- Academic
- Government agency
- Pharmaceutical company/industry
- Durable medical equipment/medical device industry
- Charitable foundation
- Other industry (food production, supplement production, etc)
- Consulting or contract research organization
- Health care service provision
- Other:

B2. Study question 1 (primary study question)
State the primary causal/associational question in this study as stated in the paper. If there are multiple questions that are equally emphasize, the use the first one written in the abstract / introduction as study question 1, the second as 2, etc. (3 sentence max)

B2a. State the exposure of interest for the study question noted in B2

B2b. State the outcome of interest for the study question noted in B2

B2c. State the point estimate and confidence interval for the reported association between the exposure and outcome for the study question noted in B2.
If more than one point estimate is reported (e.g. exposure with multiple levels), report all and label each as appropriate. If a confidence interval is not reported, report other measures of uncertainty (p-value or standard deviation).

B3. Study question 2 (if applicable)
State an additional causal/associational question in this study (i.e. "This study estimates the impact of X on Y" or "relationship between X and Y") (1 sentence max)

B3a. State the main effect/relationship reported by the article (effect estimate, confidence interval/SD/p-value).

B4. Study question 3 (if applicable)
State an additional causal/associational question in this study (i.e. "This study estimates the impact of X on Y" or "relationship between X and Y") (1 sentence max)

B4a. State the main effect/relationship reported by the article (effect estimate, confidence interval/SD/p-value).

B5. Basic study design

- Randomized controlled trial
- Observational prospective cohort
- Observational case-control
- Observational cross-sectional
- Other:

B6. What is the main sample size of the study?
If unclear and/or there are multiple sample sizes, use the sample size reported for the primary question.

Section C: Generalizability

For this section, consider an appropriate target population for which this study would be applicable. For example, if the study was on birth control, the appropriate population might be women of childbearing age.

C1. Describe the target population for the study
(1 sentence max)

C2. Describe where and among whom the sample was collected/recruited (state if the sample is not clearly defined)
(1 sentence max)

C3. Does the composition of the population sample influence the magnitude and/or direction of the results, as compared to in an ideally generalizable population?

Consider issues such as demographics, location, socioeconomic status, health status, etc.

- Yes / most likely
- Maybe / plausible
- No / implausible
- Can't be determined

C3a. If "yes," or "maybe," briefly explain the generalizability concern.
(3 sentence max)

C4. Were analyses done to help with transporting results to other populations, such as modification assessments or subgroup analyses?

- Yes
- No
- Can't be determined
- N/A

C5. Does the study administration and data collection environment influence the magnitude and/or direction of the results, as compared to a real-world change in the exposure of interest?
Consider any effects related to what might be different about the study environment, such as the impact of measurement frequency, observation effects, treatment procedure stringency, etc. Do NOT consider sample selection issues.

- Yes / most likely
- Maybe / plausible
- No / unlikely
- Can't be determined
- N/A

C5a. If "yes," or "maybe," briefly explain the concern.
(3 sentence max)

C6. Comments
(3 sentence max)

Section D: Selection bias/missing data

Answer all questions about the primary study question. Note: here, "selection bias" refers to issues affecting internal validity only.

D1. If this was a prospective study, what was the magnitude of loss to follow up?
If there are multiple endpoints, use the loss to follow-up at the main end point from the primary results.

- 0% (none)
- <10%
- 10-30%
- 31-50%
- >50%
- Can't be determined
- N/A

D1a. If applicable, how did the authors attempt to remedy this loss to follow up?

- Complete case analysis
- Survival analysis
- Censoring weights
- Methods specifically for addressing attrition (e.g. bounding, Heckman model, etc.)
- Other (describe in D4)
- N/A

D2. If data from cases and controls are generated from different sampling methods or populations, could control selection be inappropriately related to the exposure?
Does the procedure which selects controls lead to higher/lower levels of exposure in the control population than that which would be expected in the relevant general population?
- Yes / most likely
- Maybe / plausible
- No / unlikely
- N/A

D2a. If "yes," or "maybe," briefly explain the concern.

D3. What is the total magnitude of missing data for the exposure, outcome, or included covariates?
Compare to the total population as if there were no missing data in the exposure, outcome, and covariates. If missing data were primarily from certain variables, note it in the comments.
- 0% (none)
- <10%
- 10-30%
- 31-50%
- >50%
- Can't be determined

D3a. If applicable, how would you describe the missing data?
- Missing completely at random (MCAR, missing data is a simple random sample of full data)
- Missing at random (MAR, missing data is a simple random sample of full data within strata of observed data missing data can be predicted by observed data)
- Missing not at random (MNAR, missing data is stratified random sample of full data in stratum of unobserved data, or missing data cannot be predicted by observed data)
- Can't be determined
- N/A

D3b. If applicable, how did the authors remedy this missing data?
- Complete case analysis
- Multiple imputation
- Weighting
- Can't be determined
- N/A
- Other (describe in D4)

D4. Comments / describe potential bias (including direction of bias)
(3 sentence max)

Section E: Exposure

This section should be completed for the primary study question.

E1. Was the exposure/intervention randomly assigned?
- Yes
- No
- Can't be determined

E2. If not randomly assigned, is there likely to be exposure measurement error/misclassification?
- Yes / very likely
- Maybe / plausible
- No / unlikely
- Can't be determined
- N/A (randomly assigned)

E2a. If measurement error/misclassification is plausible, could it be differential with respect to the outcome?
- Note any issues in E4.

- Yes / very likely
- Maybe / plausible
- No / unlikely
- Can't be determined
- N/A (randomly assigned / measurement error and/or misclassification is unlikely)

E3. Are the pathways to changing levels of the exposure of interest relevant to the effect being estimated?
For example: In a study comparing BMI levels and the risk of an outcome, can we confidently assume that how a person changes their BMI (diet, exercise, etc) has no impact on the risk of the outcome under study?

- Yes / very likely
- Maybe / plausible
- No / unlikely
- Can't be determined
- N/A (exposure is not reasonably changeable)

E4. Comments / describe potential bias (including direction of bias)
(3 sentence max)

Section F: Outcome

This section should be completed for the primary study question

F1. Is there likely to be outcome measurement error/misclassification?

- Yes / very likely
- Maybe / plausible
- No / unlikely
- Can't be determined

F1a. If misclassification is plausible, is it the measurement error / misclassification likely to be differential with respect to the exposure?

- Yes / very likely
- Maybe / plausible
- No / unlikely
- Can't be determined
- N/A (outcome misclassification / measurement error is unlikely)

F2. Comments / describe potential bias (including direction of bias)
(3 sentence max)

Section G: Covariates

This section should be completed for the primary study question

G1. Are there concerns about bias with respect to measurement error and/or misclassification of the covariates included?

- Yes / very likely
- Maybe / plausible
- No / unlikely
- Can't be determined

G1a. If yes, describe potential bias due to covariate measurement error (including direction)
(3 sentence max)

G2. How did the authors choose covariates included in the analysis?
(check all that apply)

- ☐ Directed acyclic graph (DAG)
- ☐ Prior knowledge (without DAG)
- ☐ "Kitchen sink" (include all potentially relevant/measured variables)
- ☐ P-value based model selection
- ☐ Change-in-estimate approach
- ☐ No covariates included
- ☐ Can't be determined
- ☐ Other (describe in G4)

G3. Are there variables missing from the analysis that could introduce confounding?
Consider any factors in which persons with different levels of exposure would also have different levels of the outcome which are not included or appropriately accounted for in the model. This can include health status, socioeconomic status, demographics, geography, behavioral factors, genetics, personality, etc.

- ○ Yes / very likely
- ○ Maybe / plausible
- ○ No / unlikely
- ○ Can't be determined

G3a. If yes, list unmeasured confounders as noted by the authors and the potential for bias, including direction.
List up to 5 of the most important confounding/omitted variables. If the authors do not discuss direction of bias for these variables, include your analysis of the bias in G4. (5 sentences max)

G3b. If yes, list unmeasured confounders not noted by the authors (i.e. in the opinion of the reviewer) and the potential for bias (including direction)
List up to 5 of the most important confounding/omitted variables and 3 sentence max description. (5 sentences max)

G4. Additional comments
(5 sentences max)

Section H: Statistical methods

This section should be completed for the primary study question

H1. What was the primary analytical method(s) used?

- ☐ Standard correlation assessment (regression, t-test, chi-squared, etc.)
- ☐ Hierarchical and/or longitudinal regression models
- ☐ G-computation
- ☐ Marginal structural model (i.e. inverse probability weighting)
- ☐ Instrumental variable
- ☐ Difference in difference
- ☐ Regression discontinuity
- ☐ Interrupted time series
- ☐ G-estimation of structural nested models
- ☐ Can't be determined
- ☐ Other (describe in H6)

H2. Is the analytic method appropriate for identifying a causal relationship between the exposure and outcome in the context of this study?
Consider only the study context and data structure, ignoring issues addressed in previous sections. For the purposes of this question, the dataset structure is part of the appropriateness of the method. For example, if the question can not feasibly be answered with simple regression in a cross-sectional dataset, this may be

considered an inappropriate method, even in the case that the authors did the best possible analysis with cross-sectional data.

- Yes / very appropriate
- Maybe / unsure if appropriate
- No / inappropriate
- Can't be determined

H2a. If inappropriate or unsure, briefly describe why.
(3 sentence max)

H3. What measures of uncertainty are reported?
p-values

- Confidence intervals
- Standard errors/standard deviations
- None
- Other (describe in H6)

H4. Do statistical methods appropriately estimate error bounds?
In other words, are clustering, robust standard errors, etc. used as appropriate? This question assumes that the method and data structure used are appropriate for the question of interest.

- Yes / very appropriate
- Maybe / unsure if appropriate
- No / inappropriate
- Can't be determined

H5. Describe any robustness checks against potential flaws that were completed, including functional form assumptions and other sensitivity analyses
(3 sentence max)

H6. Comments / describe potential bias (including direction of bias)
(3 sentence max)

Section I: Summary assessment

Complete this section by imagining the "ideal RCT" for causal identification of the main study question. This RCT would ignore issues of funding, ethics, and physics, as if you were able to create different counterfactual universes to precisely determine causal identification of the study question.

I1. Please rate the overall severity of potential issues related to the following topics as compared to an "ideal RCT" with regard to causal identification.
Refer back to previous pages as necessary.

	Very high severity	High severity	Moderate severity	Low severity	Very low severity
Section C) Generalizability	O	O	O	O	O
Section D) Selection bias/missing data	O	O	O	O	O
Section E) Exposure assessment	O	O	O	O	O
Section F) Outcome assessment	O	O	O	O	O
Section G) Covariate assessment	O	O	O	O	O
Section H) Statistical methods	O	O	O	O	O

I2. What direction do you believe the net direction of bias in this study is most likely to be, considering only internal validity?
I.e. positive or negative bias, NOT over/underestimate as compared to the null

- Positive bias
- Negative bias
- Unknown direction of bias

- No/very little bias
- Can't be determined

I3. Is this study likely to be an over / underestimate of the true effect vs. the null hypothesis, considering only internal validity?

- Overestimate
- Underestimate
- No/very little bias
- Can't be determined

I4. What direction do you believe the net direction of bias in this study is most likely to be, considering both internal and external validity?
I.e. positive or negative bias, NOT over/underestimate as compared to the null

- Positive bias
- Negative bias
- Unknown direction of bias
- No/very little bias
- Can't be determined

I5. Is this study likely to be an over / underestimate of the true effect vs. the null hypothesis, considering both internal and external validity?

- Overestimate
- Underestimate
- No/very little bias
- Can't be determined

I6. Given all of the above, please rank the overall strength of causal evidence of the main results from this study.
Note that this section assumes that the study question is measuring a causal relationship.

- Very high. Results are extremely likely to approximate the results of the ideal RCT / "true" effect. Study is well-documented, contains no substantial methodological issues, is highly generalizable, and has extremely low risk of bias. Results from the study should be considered gold-standard, and are fully applicable in health policy and practice.
- High. Study is generally very well conducted and reported. Some minor methodological or reporting issues may be present, but the impact on the main results of the study is likely to be low overall. Results from the study should be considered conclusive, and utilizable in health policy and practice.
- Moderate. Some relatively mild methodological issues are present, but the overall results are likely to be similar to an ideal RCT and/or result in a conservative of the true effect. Policy and practice may consider this evidence cautiously, but more study is needed.
- Low. One or more substantial methodological issues are present in this study, particularly those that overestimate the effect size. Results should be considered hypothesis-generating, and should not be used alone in policy and practice.
- Very low. Study is at a severe risk of bias due to methodological issues. Study should not be considered in policy and practice.

I7. Please give a very brief justification for your rating.
Text will be used to generate a consensus statement on the quality of evidence for this article. (3 sentences max)

I8. Comments / questions for other reviewers
If you have any additional comments or questions for other reviewers, please state them here

Section J. Language assessment (scientific article)

For this section, consider ONLY the language included in the title, abstract, introduction, conclusion, and discussion sections (or equivalent), i.e. not methods.

J1. How strongly did authors state their research QUESTION as causal?

- Strong causal language used ("is caused by", "is due to", statements without room for doubt)

- Moderate causal language used ("may be caused by", "seems to result in", causality is suggested but with doubt)
- Weak causal / or only associative language used ("is associated with", "is correlated with", "is related to")

J2. How strongly did authors state their RESULTS as causal?

- Strong causal language used ("is caused by", "is due to", statements without room for doubt)
- Moderate causal language used ("may be caused by", "seems to result in", causality is suggested but with doubt)
- Weak causal / or only associative language used ("is associated with", "is correlated with", "is related to")

J3. Please give an example of the strongest causal phrase used in the article with respect to the main results.

J4. Does the level of technical causal language used match the quality of evidence?

- Yes (Quality of evidence and language both strong, both moderate, or both weak)
- No, causal language used is too strong for quality of evidence
- No, causal language used is too weak for quality of evidence
- Can't be determined

J5. Comments / questions for other reviewers

If you have any additional comments or questions for other reviewers, please state them here (3 sentence max)

Section K: Media article #1 assessment

For this section, consider both the research question claimed in the research article and the media article. Ignore any discrepancies between the strength of causal claim, as this will be addressed in a later section.

K1. What is the main research question stated in this article?

State the main relationship as described in this media article (i.e. "This study estimates the impact of X on Y" or "relationship between X and Y") (1 sentence max)

K2. Does the main relationship question from the media article match one of the relationships addressed in the scientific article?

- Yes
- Partially
- No
- Can't be determined

K2a. If not "yes," what is the difference between the main relationship questions in the scientific and media articles?

(3 sentence max)

K3. Does the main result claimed in the media article match results from the scientific article?

- Yes
- Partially
- No
- Can't be determined

K3a. If not "yes," what is the difference between the results claimed in the scientific and media articles? Include omission of measures of uncertainty, if relevant.

(3 sentence max)

K4. Does the media article make conclusions about a population other than the study population?

- Yes
- No
- Can't be determined

K5. Does the media article make claims about an intervention that is not the one studied?
(for example, the scientific study looked at a compound contained in some foods, but the media article reports that those foods prevent cancer)

- Yes
- No
- Can't be determined

K6. Comments / describe any unjustified claims
(3 sentence max)

Section L: Language assessment (media article #1)

L1. How strongly did the media article state the research QUESTION as causal?

- Strong causal language used ("is caused by", "is due to", statements without room for doubt)
- Moderate causal language used ("may be caused by", "seems to result in", causality is suggested but with doubt)
- Weak causal / or only associative language used ("is associated with", "is correlated with", "is related to")

L2. How strongly did the media article state the RESULTS as causal?

- Strong causal language used ("is caused by", "is due to", statements without room for doubt)
- Moderate causal language used ("may be caused by", "seems to result in", causality is suggested but with doubt)
- Weak causal / or only associative language used ("is associated with", "is correlated with", "is related to")

L3. Please give an example of the strongest causal phrase/sentence used in the article with respect to the main article results

L4. Does the level of technical causal language used match the level of technical causal language used in the scientific paper?

- Yes (causal language in both articles is strong, both moderate, or both weak)
- No, causal language used in media article is stronger than in scientific paper
- No, causal language used in media article is weaker than in scientific paper
- Can't be determined

L5. Comments / questions for other reviewers
If you have any additional comments or questions for other reviewers, please state them here (3 sentence max)

Sections M-R

Repeats Sections K and L for each associated media articles #2-4
