# Supplementary material for: Causal language and strength of inference in academic and media articles shared in social media (CLAIMS): A systematic review
Source: PLoS One. 2018 May 30;13(5):e0196346. doi: 10.1371/journal.pone.0196346 (PMC5976147; doi:10.1371/journal.pone.0196346)
Supplement: S2 File — This document contains the pre-registered protocol as registered with PROSPERO on August 5, 2016. The PROSPERO registration can be found at http://www.crd.york.ac.uk/PROSPERO/display_record.php?ID=CRD42016045197. The full, detailed protocol is available on metacausal.com/CLAIMS/protocol. (PDF) [file pone.0196346.s008.pdf]

PROSPERO International prospective register of systematic reviews

Health media review project

Emily Smith, Kathryn Andrews, Robin Audy, Winnie Bell, Alana Brennan, Alexander Breskin, Noah Haber, Jeremy Kane, Mahesh Karra, Elizabeth McClure, Ellen Moscoe, Elizabeth Suarez

Citation

Emily Smith, Kathryn Andrews, Robin Audy, Winnie Bell, Alana Brennan, Alexander Breskin, Noah Haber, Jeremy Kane, Mahesh Karra, Elizabeth McClure, Ellen Moscoe, Elizabeth Suarez. Health media review project. PROSPERO 2016:CRD42016045197 Available from
http://www.crd.york.ac.uk/PROSPERO_REBRANDING/display_record.asp?ID=CRD42016045197

Review question(s)

1. Do health-related scientific studies reported in media articles and shared on social and traditional media:

a. Demonstrate a strong causal relationship between exposure and outcome?

b. Use language that appropriately reflects the strength of causal inference in the study?

2. Do health research-related media articles shared on social and traditional media:

a. Accurately report scientific study findings?

b. Critically assess the strength of causal findings of the scientific article?

c. Reflect the strength of language used by the authors of the scientific article?

Searches

We systematically identify widely shared media articles pertaining to single studies with a health outcome, using Facebook and Twitter social media sharing statistics generated from the NewsWhip Insights platform. The NewsWhip Insights dataset is based on a privately operated social media crawler, which has been collecting data since the beginning of 2014. The platform identifies media stories, and tracks how those stories are distributed on social media platforms. The stories are then categorized internally into non-exclusive story “types,” including a category for news. Tracking for each story continues for one month after it is published and includes statistics on how many times it is shared on social media networks (e.g. Facebook, Twitter, Reddit, LinkedIn, etc.). The dataset can be queried, using titles, time periods, and content to filter results. We will query the Insights dataset to generate a list of health news articles pertaining to new research studies published in 2015 (published from January 1, 2015 0:00 EST to December 31, 2015 11:59:59 EST).

The search terms for this query are:

(categories:2) AND ((headline_en:"health" OR summary_en:"health") AND (headline_en:"study" OR

summary_en:"study") OR (headline_en:"research" OR summary_en:"research"))

where "categories 2" corresponds to NewsWhip’s internally curated categorization for sites containing news, "headline_en" is the programmatically extracted headline of the story, and "summary_en" is the programmatically generated content in English.

Types of study to be included

Media/scientific article pairs are defined as a news media article and its associated scientific study for the screening process. Inclusion criteria are applied for each media/scientific article pair. The selection process is based on the media article title and full article and the abstract of the scientific article.

The inclusion criteria are:

1. The URL link to media article is functional at the time of the review, leading to the main media article.

2. The news media article reports primarily about the findings from a single scientific article published in a peer-reviewed academic journal.

3. The scientific article referred to in the media article is identifiable through academic library sources.

4. The news media article reports that the scientific article:

a. Has a main analysis of the form exposure (dependent variable) vs. outcome (independent variable).

b. Has a health outcome as one of its main outcomes (independent variables).

c. Has strongly related primary exposures/outcomes if multiple exposures/outcomes are

equally emphasized.

d. Is measured in a human population.

e. Is a primary study, and not a review or meta-analysis.

f. Has main results generated from a single identifiable statistical model.

5. The abstract of the scientific article:

a. Has a main analysis of the form exposure (dependent variable) vs. outcome (independent variable).

b. Has a health outcome as one of its main outcomes (independent variables).

c. Has strongly related primary exposures/outcomes if multiple exposures/outcomes are

equally emphasized.

d. Is measured in a human population.

e. Is a primary study, and not a review or meta-analysis.

f. Has main results generated from a single identifiable statistical model.

Note: 4f) and 5f) were added to the inclusion criteria list post-hoc after a case study and a journal article consisting of multiple primary sub-studies were selected into the final review based on existing inclusion criteria

Condition or domain being studied

This review is about the quality of "popular" health research studies and the way in which this health research is reported by the media.

Participants/ population

The 'population' / unit of analysis is health research studies.

Intervention(s), exposure(s)

The 'population' being study is health research studies (published in peer-reviewed journals) and corresponding media reporting on these studies.

Comparator(s)/ control

There is no comparator group. This is a descriptive analysis of 'popular' health research studies.

Outcome(s)

Primary outcomes

1) scientific article strength of causal evidence,

2) scientific article language assessment regarding strength of causal evidence,

3) media article language assessment regarding strength of causal evidence.

Secondary outcomes

None

Data extraction, (selection and coding)

Study Selection:

The screeners will assess the pair for all inclusion criteria relevant at that level. If an article fails to meet any of the inclusion criteria at a given level, a note on the reason(s) for rejection will be made, and the reviewer will move to the next article. If the reviewer determines that the study either meets our inclusion criteria or that there is not enough

information to reject at the given level, the reviewer will proceed to review the media article at the next level. The order of screening is below:

1. Media article title

a. Review for inclusion / exclusion criteria

b. If not rejected, continue to media article text level

2. Media article text

a. Review for inclusion / exclusion criteria

b. If not rejected, attempt to identify the scientific article associated with the media article

i. If scientific article identified and abstract located, continue to scientific article

abstract level

3. Scientific article abstract

a. Review for inclusion / exclusion criteria

b. If not rejected, accept the article into the final review sample and continue to data

extraction and storage

After the two primary reviewers complete their assessment of the media and scientific articles for inclusion / exclusion criteria, the screening arbitrator will reconcile the lists for items to eliminate, making the final judgement when reviewers disagree.

Data Abstraction:

Three reviewers will be randomly assigned to review each scientific article and the associated media article. Two will serve as primary reviewers and the third person (the arbitrator) will be responsible for consolidating the two independent data abstraction forms. All reviewers will be given an anonymous reviewer ID. The reviewers will not know the identities of any of the other reviewers at any time, including after the study. All communication between them will occur through the study administrators, and the file linking the reviewers' identifying information with their numerical ID will be encrypted.

The screening arbitrator from screening phase will serve as the primary administrator for quality assessment/data abstraction phase.

Risk of bias (quality) assessment
One of the primary outcomes of this study is a quality assessment of the scientific study. We have developed a review tool to assess the quality of the included studies. The primary guidance for the development of this tool is from the Cochrane Handbook for Systematic Reviews of Interventions, most notably the GRADE approach.

Strategy for data synthesis
Statistical analysis will be primarily descriptive, reporting on the distribution of responses from the 50-100 arbitrator-consolidated assessments. Associations between the responses from different questions will be assessed by simple regression methodologies, such as ordinary least squares (OLS) and logistic regression as appropriate. All analysis will be performed in R software. Code used for analysis will be made publicly available at the time of publication.

Analysis of subgroups or subsets
Subgroup analyses include:

1. Place of publication for a) scientific article and b) media article

2. Place of social media share: a) Facebook, b) Twitter

Contact details for further information
Mr Haber

Harvard T.H. Chan School of Public Health

Building 1, Room 1106A

677 Huntington Avenue

Boston, MA 02115

nah827@mail.harvard.edu

Organisational affiliation of the review
None

None

Review team
Ms Emily Smith, Harvard T.H. Chan School of Public Health
Ms Kathryn Andrews, Harvard T.H. Chan School of Public Health
Mr Robin Audy,
Ms Winnie Bell, Tufts Friedman School of Nutrition Science and Policy
Dr Alana Brennan, Boston University School of Public Health
Mr Alexander Breskin, University of North Carolina Gillings School of Global Public Health
Mr Noah Haber, Harvard T.H. Chan School of Public Health
Dr Jeremy Kane, Johns Hopkins Bloomberg School of Public Health
Mr Mahesh Karra, Harvard T.H. Chan School of Public Health
Ms Elizabeth McClure, University of North Carolina Gillings School of Global Public Health
Ms Ellen Moscoe, Harvard T.H. Chan School of Public Health
Ms Elizabeth Suarez, University of North Carolina Gillings School of Global Public Health

Anticipated or actual start date
14 July 2016

Anticipated completion date
31 October 2016

Funding sources/sponsors
No funding or sponsors

Conflicts of interest
None known

Language
English

Country
United States of America

Subject index terms status
Subject indexing assigned by CRD

Subject index terms
Humans; Mass Media; Research

Stage of review
Ongoing

Date of registration in PROSPERO
05 August 2016

Date of publication of this revision
05 August 2016

Stage of review at time of this submission	Started	Completed
Preliminary searches	No	Yes
Piloting of the study selection process	No	Yes
Formal screening of search results against eligibility criteria	No	Yes
Data extraction	No	No
Risk of bias (quality) assessment	No	No
Data analysis	No	No

PROSPERO
International prospective register of systematic reviews
The information in this record has been provided by the named contact for this review. CRD has accepted this information in good faith and registered the review in PROSPERO. CRD bears no responsibility or liability for the content of this registration record, any associated files or external websites.
